# Landscape and variation of novel retroduplications in 26 human populations

**DOI:** 10.1371/journal.pcbi.1005567

**Published:** 2017-06-29

**Authors:** Yan Zhang, Shantao Li, Alexej Abyzov, Mark B. Gerstein

**Affiliations:** 1 Program in Computational Biology and Bioinformatics, Yale University, New Haven, Connecticut, United States of America; 2 Department of Molecular Biophysics and Biochemistry, Yale University, New Haven, Connecticut, United States of America; 3 Department of Biomedical Informatics, College of Medicine, The Ohio State University, Columbus, Ohio, United States of America; 4 Department of Health Sciences Research, Center for Individualized Medicine, Mayo Clinic, Rochester, Minnesota, United States of America; 5 Department of Computer Science, Yale University, New Haven, Connecticut, United States of America; University of California San Diego, UNITED STATES

## Abstract

Retroduplications come from reverse transcription of mRNAs and their insertion back into the genome. Here, we performed comprehensive discovery and analysis of retroduplications in a large cohort of 2,535 individuals from 26 human populations, as part of 1000 Genomes Phase 3. We developed an integrated approach to discover novel retroduplications combining high-coverage exome and low-coverage whole-genome sequencing data, utilizing information from both exon-exon junctions and discordant paired-end reads. We found 503 parent genes having novel retroduplications absent from the reference genome. Based solely on retroduplication variation, we built phylogenetic trees of human populations; these represent superpopulation structure well and indicate that variable retroduplications are effective population markers. We further identified 43 retroduplication parent genes differentiating superpopulations. This group contains several interesting insertion events, including a SLMO2 retroduplication and insertion into CAV3, which has a potential disease association. We also found retroduplications to be associated with a variety of genomic features: (1) Insertion sites were correlated with regular nucleosome positioning. (2) They, predictably, tend to avoid conserved functional regions, such as exons, but, somewhat surprisingly, also avoid introns. (3) Retroduplications tend to be co-inserted with young L1 elements, indicating recent retrotranspositional activity, and (4) they have a weak tendency to originate from highly expressed parent genes. Our investigation provides insight into the functional impact and association with genomic elements of retroduplications. We anticipate our approach and analytical methodology to have application in a more clinical context, where exome sequencing data is abundant and the discovery of retroduplications can potentially improve the accuracy of SNP calling.

## Author summary

We developed an approach and performed comprehensive discovery of retroduplications from 26 human populations, utilizing whole-exome and whole-genome sequencing data. Our high-resolution landscape of retroduplications reveals that variable retroduplications are effective markers of human populations and can track population divergence. We observed that novel retroduplications come from genes with relatively high expression level and co-inserted L1 elements belong to young L1 families, indicating recent retroduplication activity in human migration contributing to genetic diversity. We have also detected several interesting intragenic insertion events, including SLMO2 retroduplication and insertion into CAV3, which worth further investigation for disease predisposition.

## Introduction

Retrotransposons are class I transposable elements. In retrotransposition events, they are first transcribed into RNA and then reverse transcribed back into DNA, which are eventually inserted into a new position in the genome. It has been found that L1 retrotransponsons, the only autonomous mobile elements in human genome, also occasionally pick up cellular mRNAs as templates for reverse transcription and insertion [1–3]. Although RNA-mediated retroduplication is less common and widespread than DNA-mediated duplication [4], recent studies have revealed extensive retroduplication polymorphism in human genomes [5–7].

Retroduplication of genes contribute to new gene generation and genome evolution [4,8,9]. While most of the retroduplications suffer from lack of promoters, 5’ truncation, mutations, inactive local chromatin environment and other unfavorable factors that hinder the expression of functional protein products, they do exhibit functional impact at times. In some cases, cellular environment change, such as cancer initiation, can “activate” retroduplications, and both transcription and translation evidence of such cases have been observed [10–12]. In other cases, transcription products play a role in the expression regulation of their parent genes [13,14]. Two known transcriptional level regulatory mechanisms are RNA interference [15–17], and transcription products serving as competitive miRNA binding targets [18,19]. Sometimes retroduplications can have high impact on genomic functions when inserting into functional regions. Studies have confirmed cases in which germline intragenic retroduplications result in liver cancer susceptibility [20] and primary immunodeficiency [21]. Besides germline events, a number of studies have reported massive somatic retroduplication events and their critical roles in tumor development [20,22–25] and neuron development [26,27].

Retroduplications carry several distinctive features: exon-exon junctions, genome locations distant to parent genes, poly-A tails, and L1 transposition markers such as target-site duplications (TSDs) and human L1 endonuclease preferential cleavage sites. In this study, we developed an integrative approach to exploit these features for novel variable retroduplication identification, and successfully applied it to 2,535 individuals from 26 populations sequenced by the 1000 Genomes Project Phase 3 [28–30]. Our study adds an additional category of genetic variation to the released Phase 3 categories [29,30]. We further performed extensive population genetic analysis, association analysis, event mechanism inference, and functional analysis of retroduplications. Our study is indicative of human migration and evolution history, and provides valuable insight into retroduplications' functional impact and their association with genomic elements.

## Results and discussion

**Fig 1:**
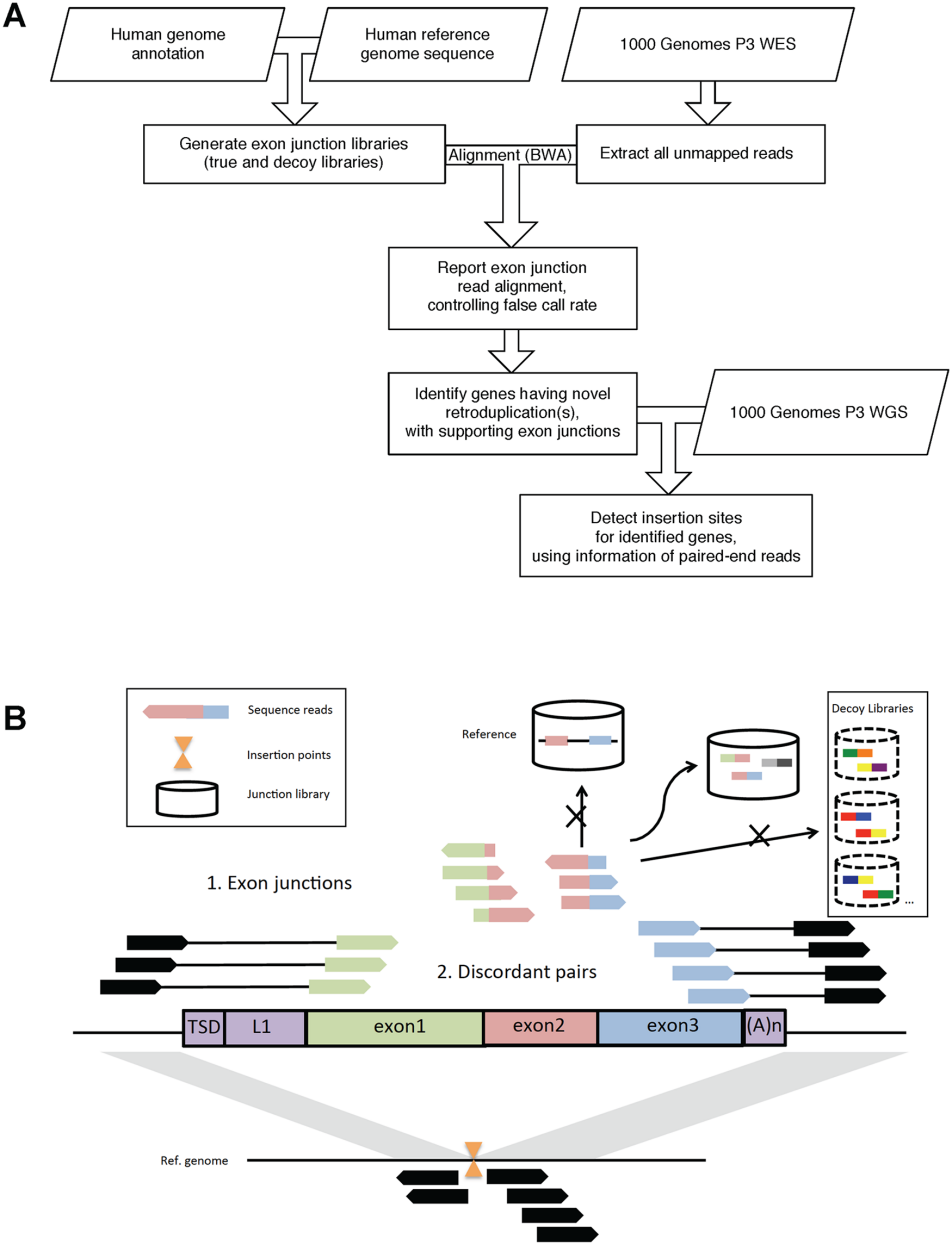
Overview of the retroduplication calling pipeline. A—A simplified flow chart of our calling pipeline. B—A schematic diagram of our strategies. We first align unmapped reads to exon junction libraries and use decoy libraries to control the false discovery rate (FDR). Then, we collect discordant paired-end reads, and cluster the reads that are mapped distal to the parent genes. Clustered distal reads indicate retroduplication insertion site.

First, we performed retroduplication discovery for each individual, using the exon-exon junction strategy on high-coverage whole-exome sequencing (WES) data (see **Supplementary Methods**, and Fig 1). We controlled the false discovery rate (FDR) using decoy exon junction libraries. As a result, we have called a total of 15,642 retroduplications from 2,533 individuals (with two outlier samples excluded) for 503 unique parent genes (**Figs A**, **B** in S1 Text; **Table A** in S1 Text; S2 Text). On average, an individual has 6 novel retroduplications identified based on exon-exon junctions. Next, we identified retroduplication insertion sites for 152 of the parent genes based on discordant paired-end reads, using shallow-sequenced whole-genome sequencing (WGS) data pooled by population (Fig 1; S3 Text). Multiple genomic features are exploited in this pipeline, in order to achieve high sensitivity in calling, while conservatively controlling the false discovery rate. The retroduplications identified in our study adds an additional category of genetic variation to the released Phase 3 categories [29,30].

Compared to previous studies of human germline retroduplications, which relied on about 1,000 shallow-sequenced individuals [5–7] from the 1000 Genomes Project Phase 1 [31], the population set and sequencing coverage in Phase 3 has scaled up the data about 10-fold combined (**Fig C** in S1 Text). Besides the retroduplication calls shared among callsets, there are also large number of calls unique to our callset, which is likely due to newly enrolled populations in Phase 3 data, and the higher sensitivity of our methods. We resolved 152/503 (30.2%) insertion sites for our predicted retroduplications, a percentage higher than previous studies [5,7]. Functional enrichment analysis for the 503 unique parent genes shows the most enriched functions are related to ribosome/structural molecule activity, intracellular organelle lumen/nucleoplasm, and protein complex assembly. This observation is in accordance with previous study [5], indicating retrotransposition is coupled with cell division.

We have identified novel retroduplications, which are insertions relative to the reference genome. There are also retroduplications that are deletions relative to the reference genome (i.e. absent in the individuals but present in the reference genome). These events can be detected by overlapping known processed pseudogenes in the reference genome with 1000 Genomes Phase 3 deletion set. We carried out this in the supplement, finding 50 such deletion events (S4 Text). This type of events is far less common than retroduplication insertions, thus we suggest focusing on retroduplication insertions in the study.

The high-resolution landscape of germline retroduplication polymorphism presented by our callset gives us the power to perform extensive analyses of retroduplication variation. Among all 503 parent genes with novel retroduplications, 361 (71.8%) are exclusively identified in a single population, while only 29 (5.8%) are commonly identified in more than 10 populations (**Fig B** in S1 Text). Retroduplications are larger events than SNPs. It is known that individual structural variations are more likely to lead to phenotypic differences than individual SNPs [32]; thus, retroduplications might be more influential and population-specific than SNPs. From all identified parent genes, we found 43 that can differentiate superpopulations (with significantly large fixation index *F*_*ST*_, adjusted empirical p-value < 0.001; see **Table B** in S1 Text).

**Fig 2:**
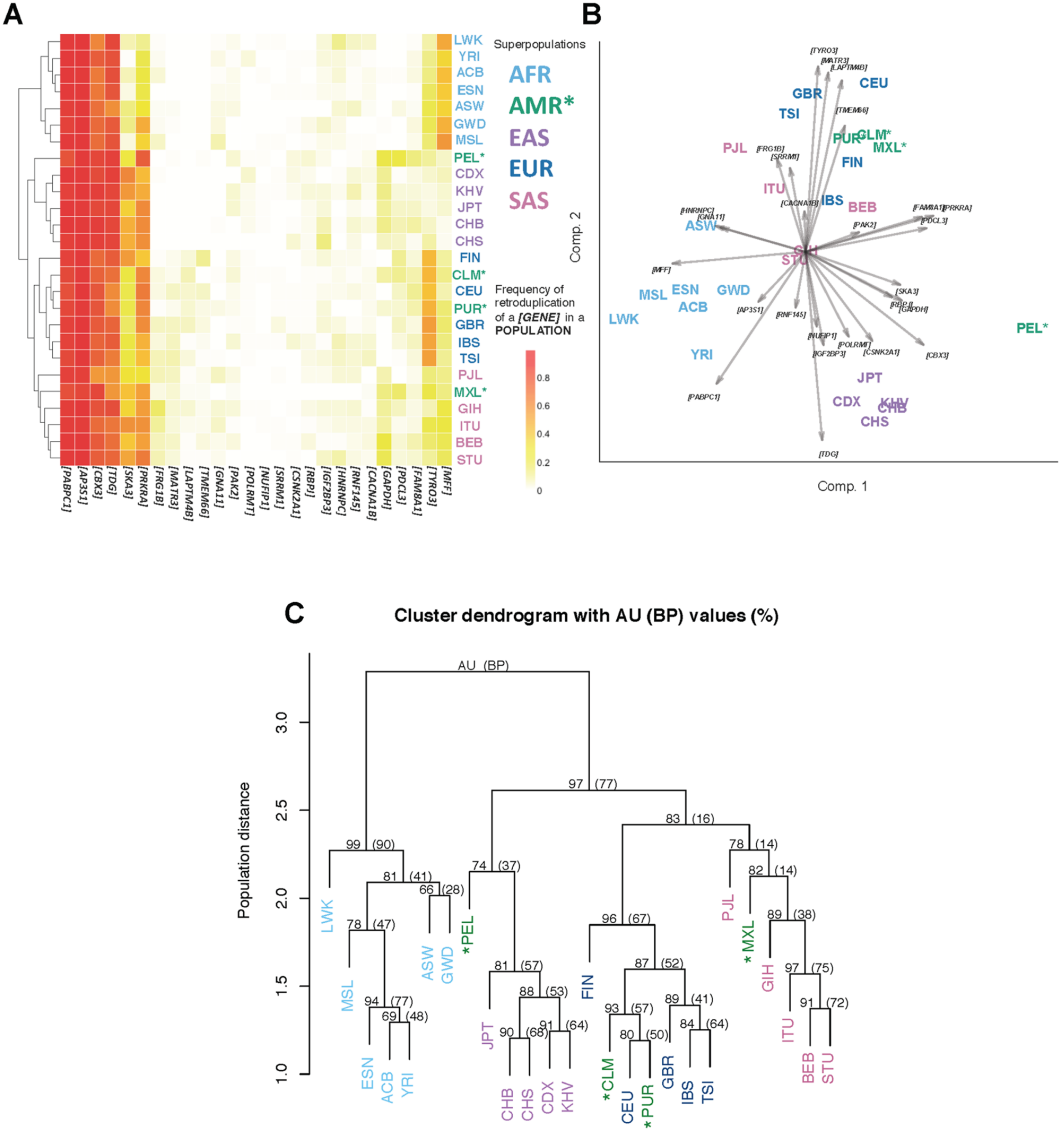
Common retroduplication frequency spectrum and phylogenetic tree. A—Frequency spectrum of 29 retroduplication events that are detected in more than 10 populations. Hierarchical clustering. B—PCA biplot of the populations based on frequencies of these 29 retroduplication events. Different colors indicate five superpopulations, i.e. AFR (African), AMR (Ad Mixed American), EAS (East Asian), EUR (European), and SAS (South Asian). Arrows represent loadings of parent genes. Ad Mixed Americans are marked with ‘*’. C—Consensus phylogenetic tree built based on novel retroduplications from all 26 populations enrolled in the 1000 Genome Project Phase 3. Bootstrap probability (BP) value is computed from ordinary bootstrap resampling. It is the frequency of the cluster appearing in bootstrap replicates. Approximately unbiased (AU) probability value is calculated from multiscale bootstrap resampling [33,34]. AU is less biased than BP. Bootstrap resampling was performed 1,000 times for generating the trees that are summarized in the consensus tree. Manhattan distance and average linkage was used in hierarchical clustering.

We hypothesize that many of the exclusive retroduplications emerged after population divergence. The frequency spectrum of retroduplication parent genes (Fig 2A and 2B; **Fig E** in S1 Text) implicates population relationships. We further constructed phylogenetic trees of human populations based on novel retroduplication variations (Fig 2C), from which we observed expected and confident cluster cohesion of superpopulations measured by approximately unbiased bootstrap probability (AU) [33,34] (African AU = 99%, East Asian AU = 81%, European AU = 96%, and South Asian AU = 78%). The phylogenetic trees can confidently represent the superpopulation structure and also show mixed populations (Ad Mixed American) mingling with other superpopulations. These observed population relationships are consistent with human migration history. We also compared our retroduplication set with the SNP set generated by the 1000 Genomes Project Phase 3 [29], and found that there are proportionally more novel retroduplications (78.9%) than SNPs (68.7%) private to a superpopulation (**Table C** in S1 Text). All the above suggests the effectiveness of retroduplications as population markers, as well as validates our approach to retroduplication discovery.

For each population enrolled in the Geuvadis RNA-sequencing project (i.e. CEU, FIN, GBR, TSI, and YRI) [35], we tested whether having novel retroduplication(s) is associated with the parent gene’s expression level. We did not observe any significant association from this analysis (S6 Text), i.e. no retroduplication event was identified as an eQTL. However, while comparing expression level of retroduplication parent genes to all genes, we see a weak but ubiquitous and statistically significant trend that novel retroduplications came from highly expressed genes (p-value < 1.4 × 10^−5^ for each population, calculated from omnibus tests, see S7 Text). It is consistent with our expectation that the more mRNAs a gene produces, the higher probability that it will be converted into complementary DNA and inserted back into the genome.

**Fig 3:**
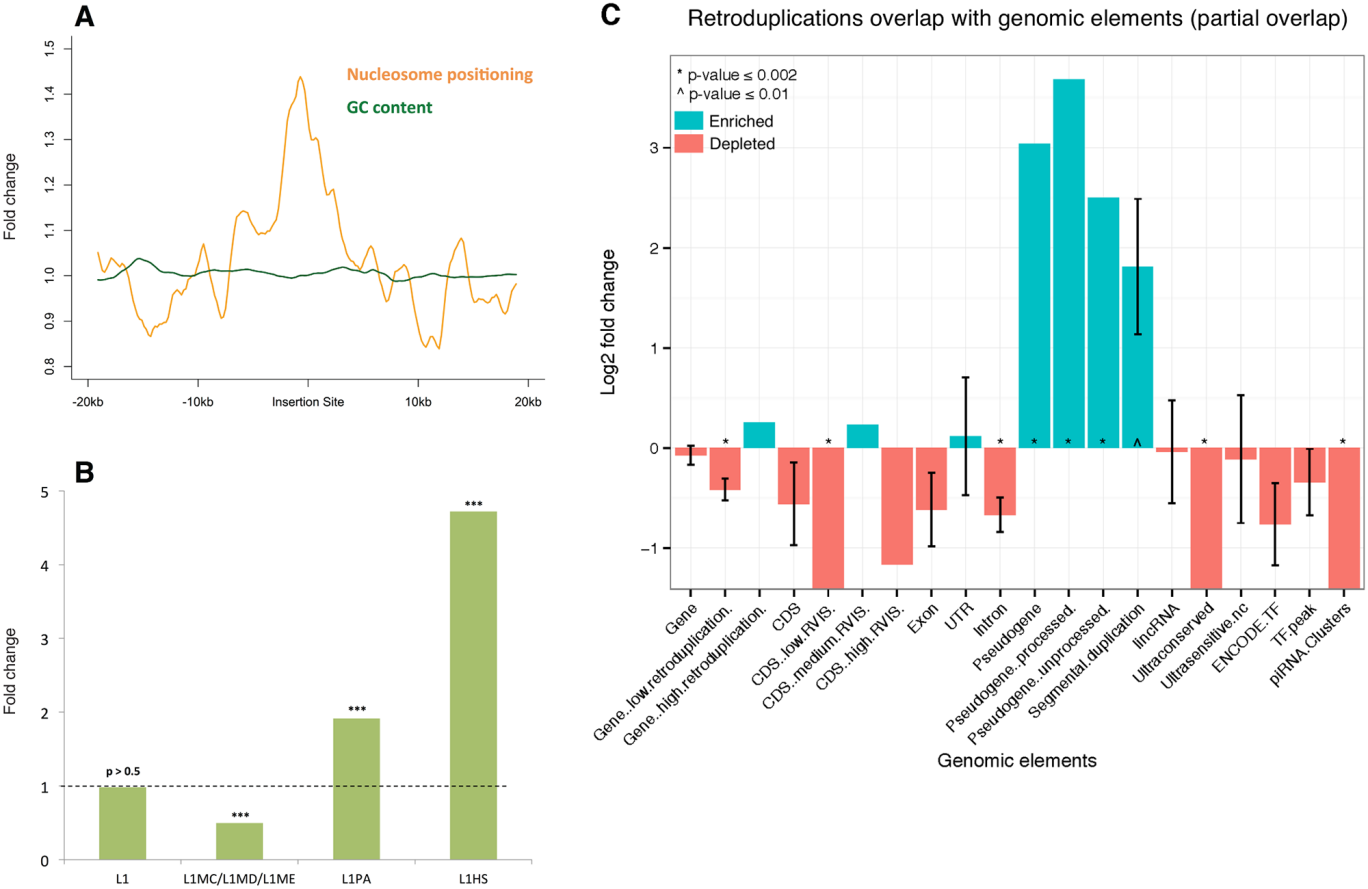
Overlap between retroduplication insertion sites and genomic features/functional elements. A—Aggregation plot around insertion sites with strongly positioned nucleosomes. B—Association between discordant read clusters that only have support on one side and L1 element subfamilies. Fold change and empirical p-values were obtained from permutations tests. *** indicates adjusted p-value < 0.001. C—Overlap between genomic elements and retroduplication insertion sites. The enrichment of overlap is expressed as log2 fold change of the observed overlap statistic versus the mean of its null distribution. Positive (negative) log2 fold change indicates enriched (depleted) genomic element-insertion overlap, compared to random background. * indicates empirical p-value ≤ 0.002.

To investigate local genomic features around insertion sites which might explain localization preference and imply retroduplication mechanism [36], we tested the association of genomic features with insertion sites. Inheritable retroduplication events occurred in germline so we focused on gametes, especially sperm. The germline mutation rate in male is higher than that in female, maybe due to the greater number and continuous nature of cell divisions in sperm formation [37–40]. We found that retroduplication insertion sites are enriched within hypomethylated regions in sperm (2.0-fold, empirical p-value < 0.0012). It is likely that retroduplication events exhibit certain preference in insertion sites associated with open chromatin. Furthermore, we characterized nucleosome positioning [41,42] around insertion sites. Overall, insertion sites show high regularity of nucleosome location (empirical p-value from permutation test 2×10^−4^) (Fig 3A). High nucleosome regularity often indicates the presence of chromatin remodeling and DNA binding proteins [43], which creates favorable loosely packed microenviroment for insertion.

Insertion points can be supported by discordant reads from both sides or just one side. We hypothesized that the insertion points with support only from a single side are the insertions with L1 element co-insertion. This is because that L1 involved in retroduplication is sometimes co-duplicated and co-inserted next to the retroduplicated segment. This type of co-insertion event can be detected by looking at the insertion sites that only have discordant read support on one side. In these cases, we found co-inserted L1 tend to belong to young L1 subfamilies, represented by L1HS (4.7-fold, p-value < 0.001) and L1PA (1.9-fold, p-value < 0.001) (Fig 3B). Contrastingly, for insertion sites without evidence for co-insertion (i.e. insertion sites that are supported by both sides) we did not observe such young L1 preference (p-value > 0.05). There is no fundamental preference for retroduplicated DNA segments to insert into other retroelements such as L1 elements. Enrichment of young and active L1 subfamilies involving in speculated L1 transductions suggests these novel retroduplication variants happened very recently.

In order to investigate the functional impact of retroduplication insertions on genomic functions, we tested the significance of overlap between retroduplication insertion sites and genomic elements compared to random genomic background (Fig 3C). As expected, ultraconserved regions are significantly depleted (p-value < 0.001). This observation is consistent with our knowledge that in general population, variable retroduplications should not interrupt with evolutionary or functionally constrained regions. Besides, we observed that intron regions are also depleted (p-value < 0.01), which might be due to negative selection that maintains conserved alternative splicing by avoiding interruption from insertion into introns. In addition, we observed segmental duplication (SD) regions to be enriched. Kim et al. [44] also found an association between SDs and processed pseudogenes, and observed significant amount of SDs flanked by matching pseudogenes. This is consistent with our observation. One reason of this association might be that the repeats generated by retroduplications are associated with non-allelic homologous recombination (NAHR) which contributes to SD formation [45–47]. It is known that NAHR is associated with open chromatin [36] and we also observed that retroduplication insertion has preference on open chromatin, which indicates open chromatin might play a role in the co-localization tendency of SDs and processed pseudogenes.

Among the 43 parent genes that differentiate superpopulations (see the top 43 genes in **Table B** in S1 Text), we have detected several potentially impactful intragenic insertion events. For example, we observed that SLMO2 (slowmo homolog 2, ENSG00000101166) is retroduplicated and inserts into the last intron of CAV3 (caveolin 3, ENSG00000182533). SLMO2 retroduplication insertion sweeps through all seven African populations almost exclusively. Based on exon-exon junction evidence, we found 30 cases in African populations and only one case in MXL (Ad Mixed American; S5 Text). CAV3 variants are strongly associated with cardiac dysrhythmia, such as long QT syndrome [48] and sudden infant death syndrome [49]. Epidemiological studies have shown that African descendant is a risk factor for prolongation of QT interval [50] and sudden infant death syndrome [51]. Such insertion events might warrant further investigation for susceptibility of diseases. We have identified a total of 12 intragenic insertion events could be related to diseases, and report the full list and affected populations (see **Table D** in S1 Text).

A final point about retroduplications: they could have an eroding effect on the correctness of SNP genotyping in parent genes or create a false image of mosaicism. We showed in a simple model that if a retroduplication carries an alternative allele, the SNP genotyping quality deteriorates significantly inside the parent gene (**Fig F** in S1 Text). We found that as the sequencing depth increases, SNP calling performance deteriorates, regardless of genotypes.

In summary, we developed an integrative approach for variable retroduplication discovery and successfully applied it to whole-exome and whole-genome sequencing data of 2,535 individuals from 26 populations. We have shown the power of leveraging high-coverage whole-exome sequencing data in retroduplication identification. Furthermore, we performed comprehensive analysis of our large retroduplication dataset, which reveals variational landscape of novel retroduplications, and shed a light on population differentiation, and functional impact of retroduplications on the genome.

## Materials and methods

### Data resources

Whole-exome sequencing and whole-genome sequencing data of 2,535 individuals from 26 populations were generated by the 1000 Genomes Project Phase 3 (whole-genome sequencing with mean depth 7.4x and read length of 100bp; targeted exome sequencing with mean depth 65.7x and read length of 76bp) [28–30]. Population description can be found at http://www.1000genomes.org/category/frequently-asked-questions/population. Protein-coding gene expression data (Peer-factor normalized RPKM) is obtained from the Geuvadis RNA-sequencing project [35], which generated RNA sequencing data from lymphoblastoid cell lines of 462 individuals from 5 populations (CEU, FIN, GBR, TSI and YRI) enrolled in the 1000 Genomes Project. We use human reference genome build 37 [52] and GENCODE v19 human genome annotation [53] in the study.

### Calling pipeline

The calling pipeline is developed and customized for generating retroduplication calls from high-coverage exome sequencing data. A simplified flowchart of the current pipeline is shown in Fig 1. We also provide the code for download (http://retrodup.gersteinlab.org).

#### Build true and decoy exon junction libraries

For calling retroduplications from whole-exome sequencing data, we need to build exon junction libraries from annotated protein-coding exons. The true exon junction library is built by joining pairs of protein-coding exon segments within the same genes, while maintaining exons’ order on the strand. Exon segments of length 100 bases adjacent to the joining splice sites are combined (**Fig D** in S1 Text). We also build five decoy exon junction libraries for the purpose of controlling FDR. The decoy exon junction libraries contain fake exon junctions, in which exon annotations are shifted by e base(s) on both sides (i.e. start location + e, end location - e). e is taken as 1, 2, 3, 6, and 12 for each decoy exon library, respectively.

#### Generate unmapped read alignments

We generate reduplication calls for each individual. Unmapped reads can be utilized for calling novel retroduplications that are absent in the reference genome. We use SAMtools [54] to extract unmapped reads from exome bam files, then use BWA-0.7.7 to align the unmapped reads to all of true and decoy exon junction libraries (**Fig D** in S1 Text). d1 and d2 are the number of bases that the read maps to either exon segment. min(d1, d2) ≥ d is required for a newly mapped read to be reported from our pipeline. We also calculate the mismatch rate r for each mapped read. d and r are parameters automatically tuned in the range [1, 15] and [0.00, 0.05], respectively, ensuring the largest number of calls from the true exon junction library while satisfying no false calls from any decoy library.

#### Estimate FDR of the exon-exon junction callset

We optimize the calling parameters so that no calls are detected in any decoy library, still this does not guarantee that the generated retroduplication calls are free of false positives. Let us assume that per sample FDR is *λ*. For simplicity, but without losing generality, we assume that *λ* is uniform across all samples. Then, the count of false calls per sample follows a Poisson distribution. The chance of having zero false calls per sample is *exp(-λ)*. Since we never detect false calls in the 2,533 samples, *exp(-λ)*^*2533*^ is the chance of observing no false calls. For 95% confidence level, this probability is equal to 0.05. This yields per sample FDR *λ* of 1.2×10^−3^. Similarly, for 99% confidence level, *λ* is 2.7×10^−3^. This projects to 3 (at 95% confidence) and 7 (at 99% confidence) false calls over the entire callset. Thus, for the 503 unique parent genes with variable retroduplications, we estimate <2% FDR with 99% confidence.

Moreover, as we always try to move further to more restricted calling criteria after no call is detected in decoy libraries, our FDR estimation above is conservative. Using additional simulated decoy libraries with different shifting coordinates as test libraries, we do not detect any false positive call under our final calling parameters. This further supports our low FDR estimation. Last, we further estimate FDR using real data (**Table E** in S1 Text).

#### Report novel retroduplication calls

Multiple “previously unmapped” reads (unmapped to the reference genome) might be mapped to the same exon-exon junction, supporting the existence of the novel exon-exon junction. Furthermore, multiple exon-exon junctions with mapped reads might support the existence of a retroduplication event. We report a gene having novel retroduplications, when it has at least two non-overlapping supporting exon-exon junctions, and at least one junction is supported by at least two mapped reads. The genes (also called parent genes) with novel retroduplications are called for each person individually. We noticed that the 1000 Genomes Project Phase 3 provides paired-end sequencing data for all individuals but NA19318. We include this individual into our analysis, as single-end sequencing does not seem to affect the performance of this pipeline.

#### Detect retroduplication insertion sites

In the insertion site detection step, we pool low-coverage whole-genome sequencing data by population, and call insertion sites for each population. We search for discordant paired-end reads (with a minimum quality score of 15) with one read correctly mapped to the parent gene, and the other read mapped to a different chromosome or at least 1 kb away from the gene. In order to mitigate false discovery, we limit our searching scope to the parent genes identified from the exon-exon junctions.

Read pairs with proper orientations are clustered using average linkage clustering. It can be shown that this linkage criterion is not likely affected by the local coverage. Assuming uniform distribution of reads, it can be shown mathematically that the expected distance between reads supporting the same insertion point is

$$\frac{2\left(IS-RL\right)+1}{3},$$

where *IS* is the insertion size and *RL* is the read length. As the insertion size in most cases is around 200–400 bp and the read length is about 70–100 bp, we choose 500 bp as the cut-off for average linkage distance to stop clustering. This cut-off not only takes the deviations of insertion size into consideration, but also allows sufficient space for target site duplications (TSDs). A valid insertion site must have at least two reads on both sides (i.e. stands). Overlapped insertion sites with identical parent gene and orientation are further merged across populations, as these sites should represent one single event.

In our insertion site detection step, we have discovered single-side clusters that have sufficient number of supporting reads. We require at least four reads on one side and no reads on the other side to call those incomplete single-side events. Single-side events across populations are merged by requiring identical parent gene, same orientation, and within 500 bp distance using locations defined by the cluster of one end. Also we only use insertion sites on chromosomes (i.e. exclude alternative locus).

#### Detect retroduplication deletions

Retroduplication deletions (relative to the reference genome) are the variable retroduplications that are absent in the individuals but present in the reference genome. We detect the retroduplication deletions by overlapping known processed pseudogenes in GENCODE v19 with 1000 Genomes Phase 3 deletion set, requiring the processed pseudogene region overlaps at least 50% of the deletion region. The results are available in S4 Text.

### Build population phylogenetic trees based on novel retroduplication calls

#### Generate retroduplication frequency matrix

Some retroduplication parent genes are called commonly among multiple populations, while some others are called exclusively in a single population. Besides, parent genes are called at different frequencies within a population. This information can be used for measuring distance between populations, while taking into account different retroduplication frequencies. We define a retroduplication frequency matrix, from which distance measures can be calculated.

Suppose there are *N* populations, and *M* unique parent genes are identified in these populations. The retroduplication frequency matrix *A* is defined as an *M×N* matrix, with each element *A*_*m*,*n*_ (*m = 1*,*2*,…,*M*; *n = 1*,*2*,…,*N*) being a value in [0, 1], representing the percentage of individuals in population *n* having this unique parent gene *m* being retroduplicated.

#### Bootstrap phylogenetic trees

We use Manhattan distance as the distance measure between each pair of populations (i.e. Manhattan distance between two columns in *A*). Average linkage is used in hierarchical clustering for generating each tree. 1,000 bootstrap replications are performed, and the uncertainty is assessed using Pvclust [33]. The reported AU (Approximately Unbiased) probability values [33,34] are used to indicate the certainty of sub-tree structures generated from multiscale bootstrap resampling [55–57]. The higher the AU probability value, the more confident the sub-tree structure is.

### Detect population differentiation due to retroduplication polymorphism

We check population differentiation due to retroduplication polymorphism, based on retroduplication frequencies in different superpopulations. Herein we pool the 26 populations into 5 superpopulations (African, Ad Mixed American, East Asian, European, and South Asian) as defined by the 1000 Genomes Project. For each given retroduplication parent gene, we calculate the population differentiation measure equivalent to the fixation index [58]. We define the test statistic

$$F_{ST}=\frac{p\left(1-p\right)-\sum_{i=1}^{5}c_{i}p_{i}\left(1-p_{i}\right)}{p\left(1-p\right)},$$

in which *i* = 1,…, 5 corresponds to the *i*th superpopulation, *p* is the retroduplication frequency of a given parent gene in the total population, *p*_*i*_ is the retroduplication frequency of the same parent gene in the *i*th superpopulation, and *c*_*i*_ is the relative population size of the *i*th superpopulation. *c*_*i*_ is calculated as the number of individuals in the *i*th superpopulation divided by the number of individuals in the total population. The larger the *F*_*ST*_, the more different the retroduplication frequencies among superpopulations. One-tailed empirical p-value is calculated comparing the observed *F*_*ST*_ versus the null distribution of *F*_*ST*_. The null distribution is calculated from 1,000 fake population sets generated by shuffling individual labels, while maintaining the size unchanged for each population. By the significance of *F*_*ST*_, i.e. the p-value adjusted by Benjamini-Hochberg procedure [59], we can detect the retroduplications that can differentiate populations.

### Analyze association between retroduplication and gene expression

We utilize our retroduplication callset and the Geuvadis gene expression data (Peer-factor normalized RPKM) [35] to analyze the association between retroduplication occurrence and gene expression. Matching data of the individuals enrolled in both the 1000 Genomes Project and the Geuvadis project are used. The association tests are performed for each population, respectively, in order to rule out the confounding by population stratification.

#### Retroduplication eQTL analysis

For a certain population, we perform the association test within the set of retroduplication parent genes: test whether having novel retroduplication(s) or not is associated with the parent gene’s expression level.

First, differential expression of each parent gene is tested between the group of individuals that have novel retroduplications of this gene and the group of individuals that do not. Two-sided Wilcoxon rank sum test is used. P-values are adjusted by Benjamini-Hochberg procedure [59]. A gene is reported to be differentially expressed in the parent gene set if its adjusted p-value is less than 0.05. Furthermore, the global differential expression of all the parent gene set is tested using Fisher’s combined probability test [60] on unadjusted p-values. This omnibus test can test the combined effect of multiple parent genes, whose individual effects are not necessarily strong. If the combined p-value is less than 0.05, we can conclude that the association between retroduplication variance and parent gene expression is significant. The results are available in S6 Text.

To re-confirm the result, we also perform two-sided Wilcoxon signed rank test. For each gene, medium expressions of both groups (having the novel retroduplication or not) are paired. The test result is consistent with that of the Fisher’s method.

#### Expression level of retroduplication parent genes compared to all genes

For a certain population, we test whether the retroduplication parent genes are highly expressed among all the genes measured in the Geuvadis dataset. We take medium expression value over all individuals for each gene as the representative expression value. One-tailed empirical p-value is calculated comparing the expression value of each parent gene versus the null distribution of expression values of all genes. It indicates the significance of each retroduplication parent gene having high expression value among all genes. Fisher’s combined probability test is performed on the empirical p-values. If the combined p-value is less than 0.05, that means in general the parent genes are significantly highly expressed among all genes. The results are available in S7 Text.

### Explore association between local genomic features and retroduplication insertion sites

To test the association between sperm methylation patterns and retroduplication insertion sites, we intersect out insertion sites with hypomethylated regions in sperm [61]. L1 annotation (RepeatMask), ENCODE HESC DNase I hypersensitive data and genomic GC contents are downloaded from the USCS Genome Browser [62]. Well-positioned nucleosome data is obtained from a recent study on multiple individuals [63].

We randomly shuffle the locations of insertion sites for 10,000 times on the same chromosome, excluding the gap regions, to obtain an empirical distribution of the null hypothesis. For fold changes, we use the mean of this distribution as the best estimate of the expected value. Calculation of p-value is empirical in order to be conservative. We use Bonferroni correction in case of multiple hypothesis testing. Unless specified otherwise, we only report corrected p-value. In order to avoid any effect of the difference of location precision across different insertion sites, we enlarge the insertion site region to 500 bp while keeping the middle point of insertions unchanged. We also exclude insertion points on alternative locus in the genome.

For aggregation plot on well-positioned nucleosome and GC content, we use 200 bp bins to calculate the base overlap, and the final plot is further window-smoothed with window size of 10. Normalization is performed by taking the mean value of the first and last 20 bins as background. We use the GC content from UCSC browser track, which is binned in 5 bp.

### Investigate impact of retroduplication insertions on genomic functions

We test the significance of overlap between retroduplication insertion sites and genomic elements, including gene, CDS, exon, UTR, intron, pseudogene and lincRNA annotated in GENCODE v19, and ultraconserved regions (evolutionary constraint regions across species), ultrasensitive non-coding regions (regions particularly sensitive to disruptive mutations) and TF (transcription factor) peak regions obtained from ENCODE RNA-seq data [10] and literature [30,64–67]. The overlap between a genomic element type and the insertion sites is measured by the partial overlap statistic, which is the count of genomic elements that have at least 1 bp overlap with the detected insertion sites.

We randomly shuffle the locations of insertion sites for 1,000 times on the same chromosome, excluding the Hg19 gap regions, to obtain an empirical distribution of the null hypothesis. In the permutation tests, the null distribution of the overlap measures is calculated from true genomic elements intersecting randomly shuffled insertion locations. The enrichment of overlap is represented by log2 fold change of the observed overlap statistic versus the mean of its null distribution. Empirical p-value is calculated.

In order to avoid any effect from different location precisions, we enlarge the insertion intervals uniformly to 1,000 bp, while keeping the middle point of insertions. We only use insertion sites on chromosomes (i.e. exclude alternative locus) in the analysis.

### Functional enrichment analysis

We use DAVID [68] to annotate functional terms for retroduplication parent genes, and survey functional term enrichment.

### Search for literature supported disease-associated insertion events

We generate a list of genes where the novel retroduplications insert into. We then search these genes in the DISEASES database [69] to find disease-gene associations reported in literature.

## Supporting information

S1 Text Supplementary file. This file contains supplementary figures and supplementary tables.
(PDF)

S2 Text Retroduplication callset derived from indicative exon-exon junctions. Retroduplication calls from each person are listed. Each row contains the following information: the junction location represented by the interval between a pair of exons being joined (Chrom: chromosome, Start: end site of the upstream exon, End: start site of the downstream exon), Parent Gene ID, the person’s ID in the 1000 Genomes Project, and the population abbreviation.
(XLSX)

S3 Text Detected retroduplication insertion sites. The file contains the information of detected insertion sites.
(XLSX)

S4 Text Detected retroduplication deletions. The file reports overlaps between deletions (DEL) and processed pseudogenes where the processed pseudogene region overlaps at least 50% of the deletion region. The first nine columns are the information for each DEL region (chromosome, start site, end site, ID, REF, ALT, QUAL, FILTER and INFO retrieved from Phase 3). The last three columns are the information for overlapping processed pseudogenes (chromosome, start site, end site).
(BED)

S5 Text Retroduplication counts and frequencies in five superpopulations. The file contains the retroduplication counts (in terms of the number of individuals having the retroduplication in a superpopulation), and the retroduplication frequencies, for all the 503 unique parent genes detected in the whole callset.
(XLSX)

S6 Text Retroduplication eQTL results. The file contains retroduplication eQTL results for five populations (CEU, FIN, GBR, TSI, YRI). Each sheet contains the result of one population. Each row (except the last) contains the following information: Parent Gene ID, the statistic from two-sided Wilcoxon rank sum test, the original p-value from the test, and the p-value adjusted by Benjamini-Hochberg procedure. The last row contains the combined p-value from the omnibus test.
(XLSX)

S7 Text Expression level of retroduplication parent genes compared to all genes. The file contains gene expression level comparison results for five populations (CEU, FIN, GBR, TSI, YRI). Each sheet contains the result of one population. Each row (except the last) contains the following information: Parent Gene ID, the observed statistic (medium of the expression level of the parent gene), quantile of the observed statistic compared to null distribution, the empirical p-value, and the p-value adjusted by Benjamini-Hochberg procedure. The last row contains the combined p-value from the omnibus test.
(XLSX)
