# Supplementary material for: Landscape and variation of novel retroduplications in 26 human populations
Source: PLoS Comput Biol. 2017 Jun 29;13(6):e1005567. doi: 10.1371/journal.pcbi.1005567 (PMC5510864; doi:10.1371/journal.pcbi.1005567)
Supplement: S1 Text — This file contains supplementary figures and supplementary tables. (PDF) [file pcbi.1005567.s001.pdf]

a)

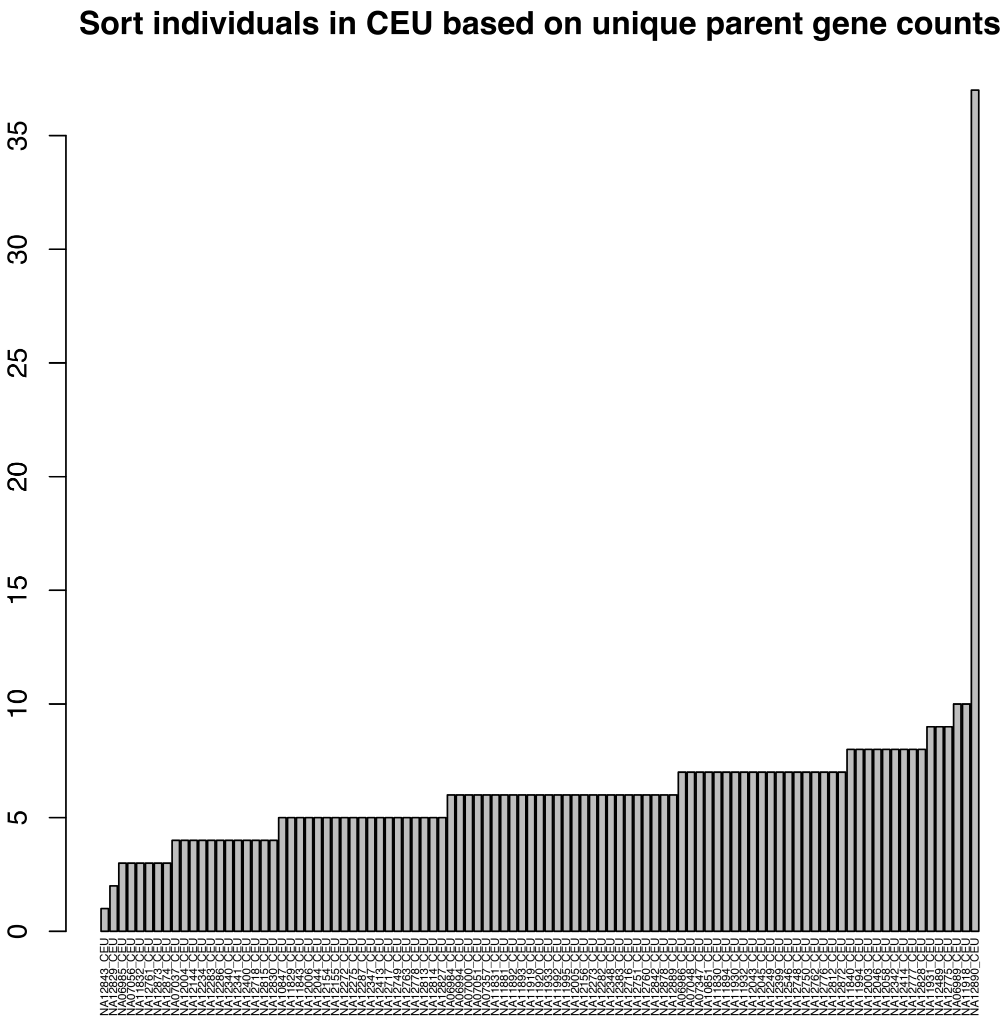
Sort individuals in CEU based on unique parent gene counts
0
5
10
15
20
25
30
35

b)

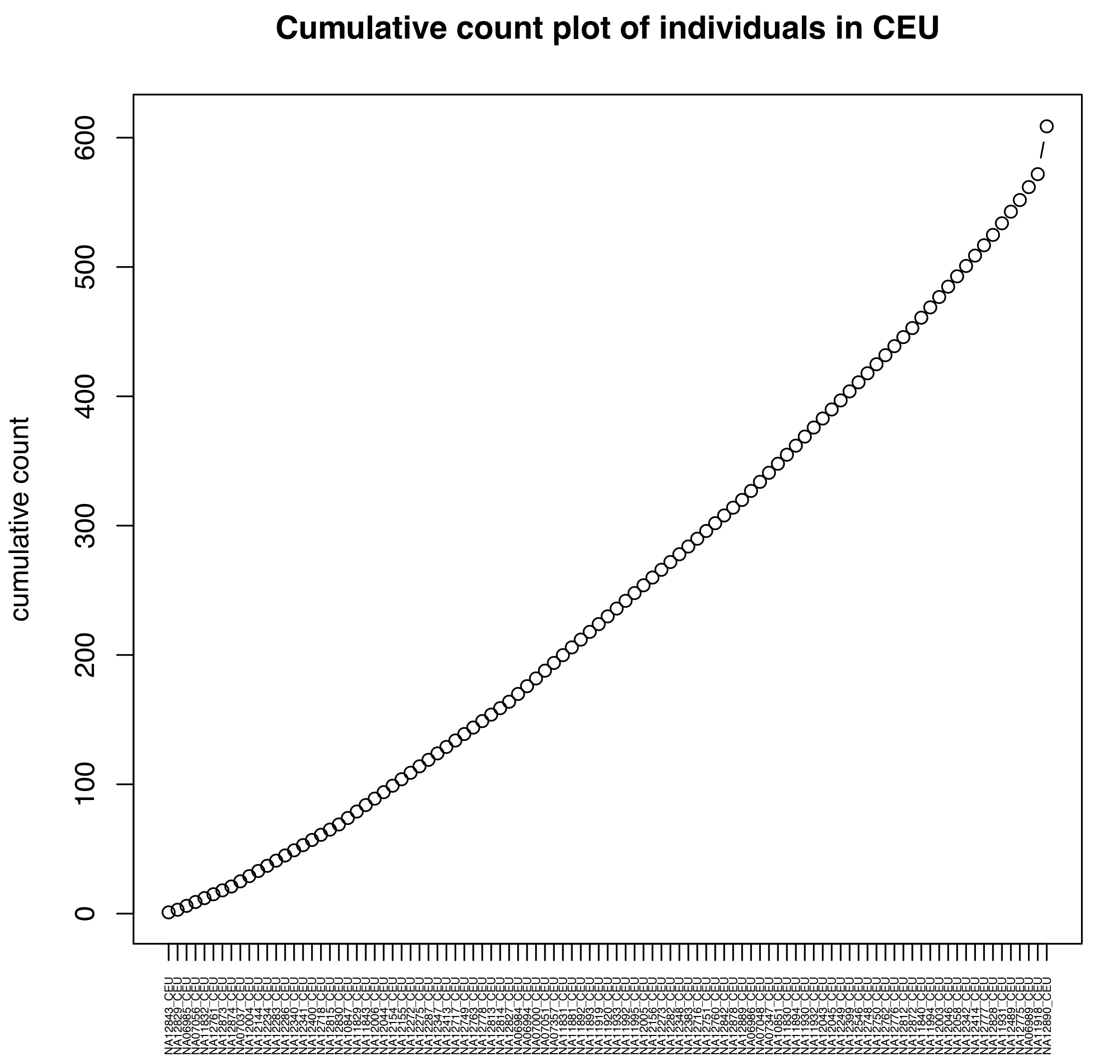
Cumulative count plot of individuals in CEU
cumulative count
0
100
200
300
400
500
600

c)

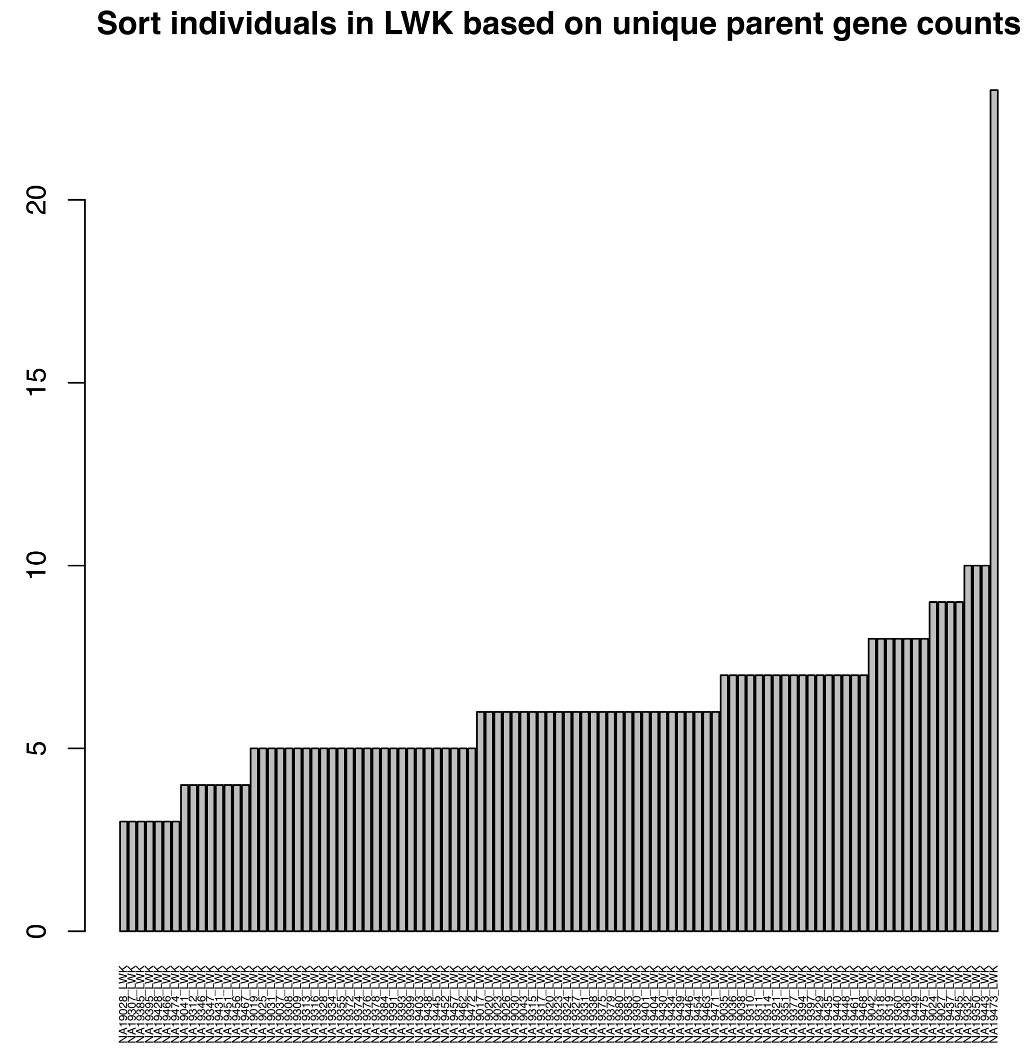

Sort individuals in LWK based on unique parent gene counts
0
5
10
15
20

d)

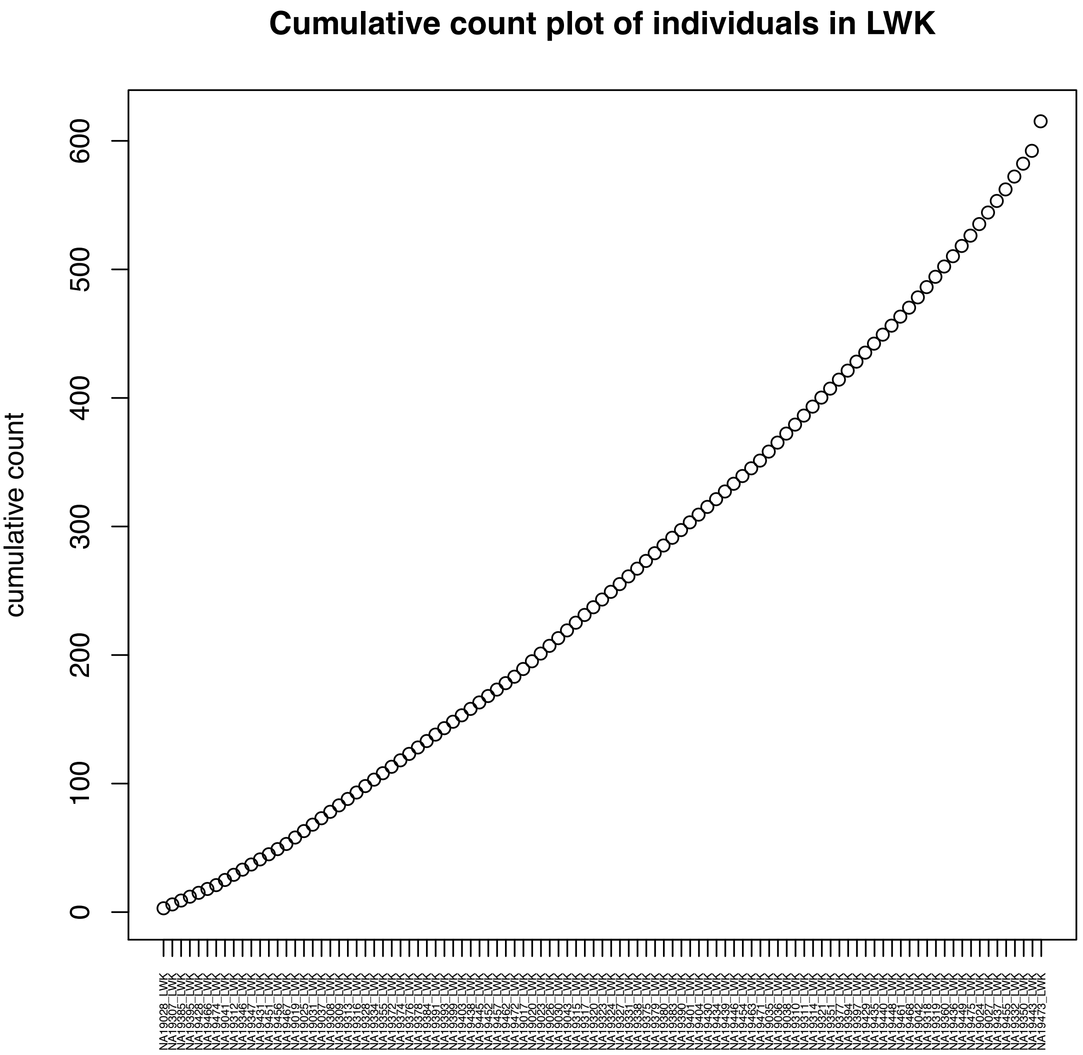

Fig A. Diagnosis plots for detection of outlier individuals that have exceptionally high number of retroduplication calls. In each population, we sort the indivuals based on the number of unique parent genes having novel retroduplications. We have detected two individuals with exceptionally high number of retroduplication calls: NA12890 in CEU, and NA19473 in LWK. Diagnosis plots of CEU (panel a, b) and LWK (panel c, d) are shown here. Individuals in each population are sorted by increasing number of retroduplication calls.

a)

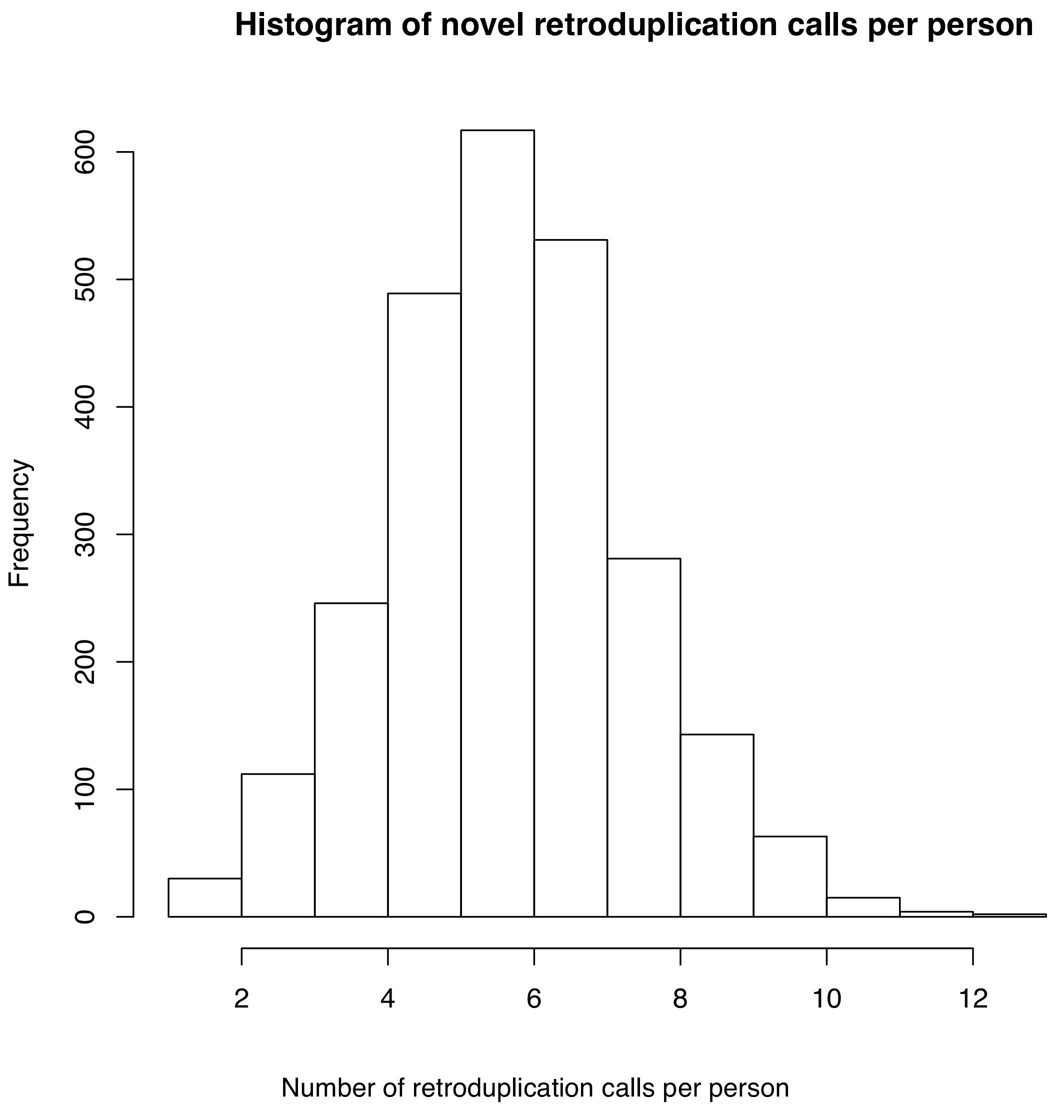

Histogram of novel retroduplication calls per person
Frequency
0
100
200
300
400
500
600
2
4
6
8
10
12
Number of retroduplication calls per person

b)

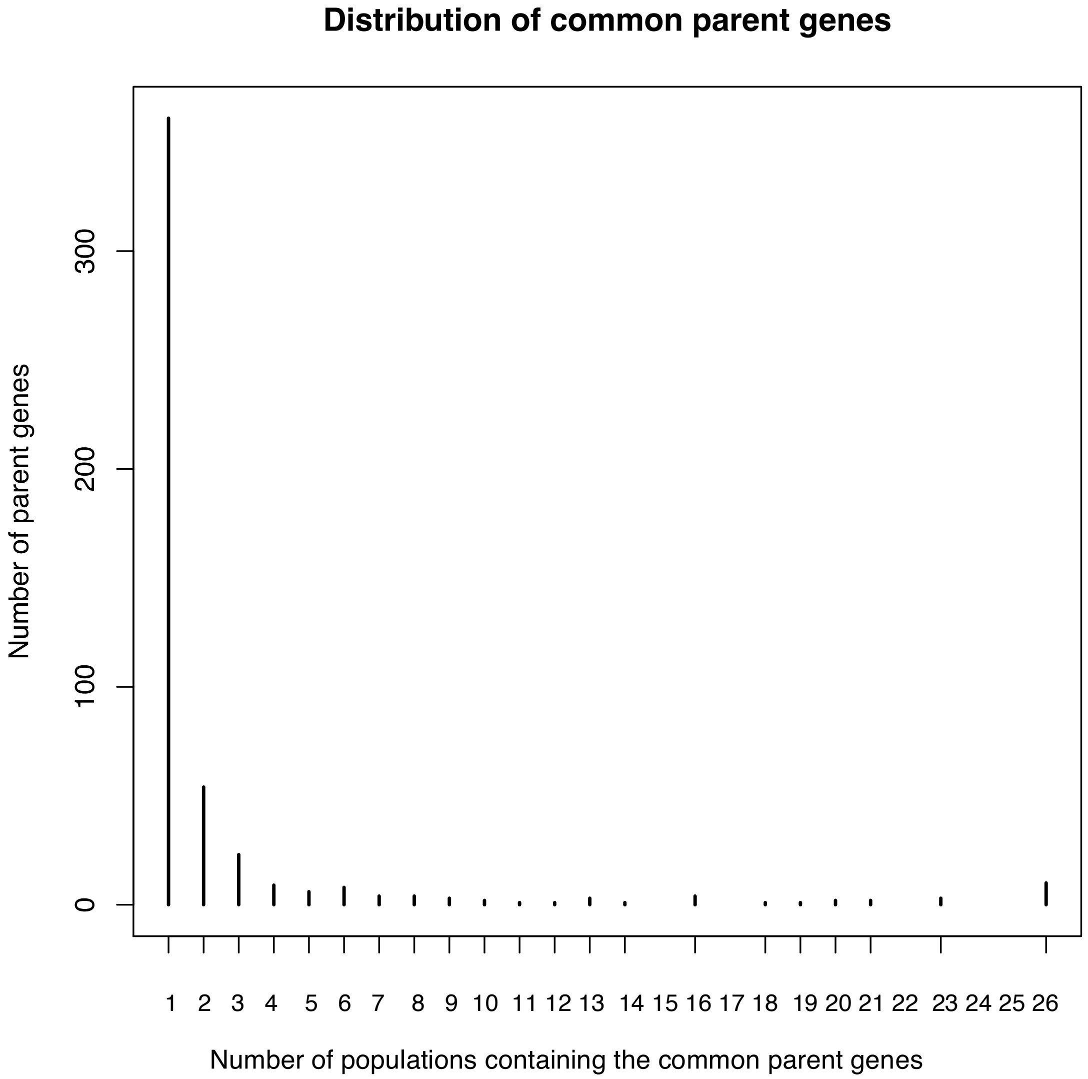

Fig B. Summary statistics of the retroduplication call set generated based exon junction libraries. a) – Histogram of novel retroduplication calls per person. b) – Distribution of common parent genes among populations. Two outlier individuals, NA12890 in CEU and NA19473 in LWK, have been excluded while generating the plots.

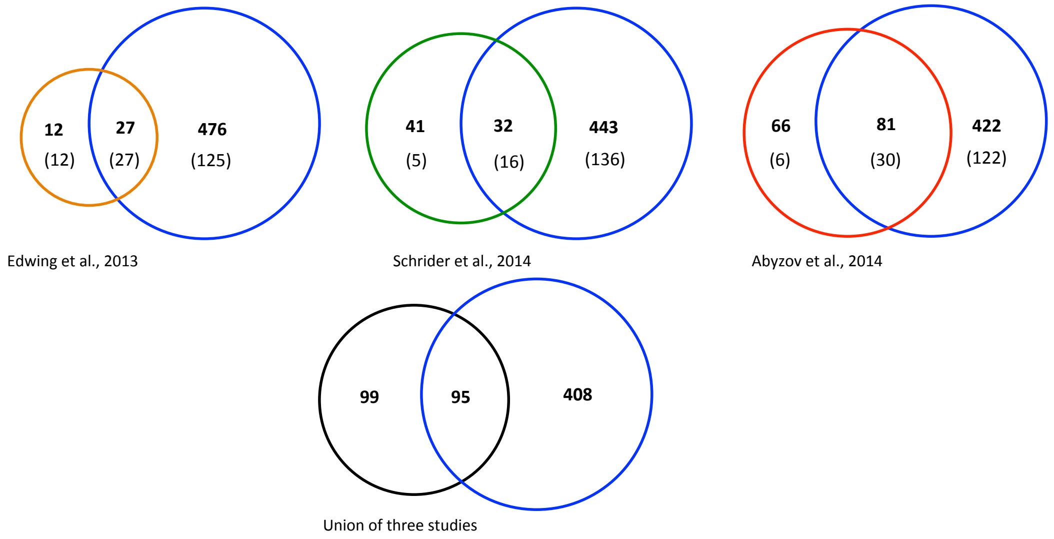

Fig C. Venn digram of callset comparison between this study and three previous studies. Concordances are called based on insertion sites if they are available in both datasets. When insertion sites are unknown, we use gene names. Numbers in parentheses are the number of insertion points in agreement. Thses circles are not drawn proportional to actual data size.

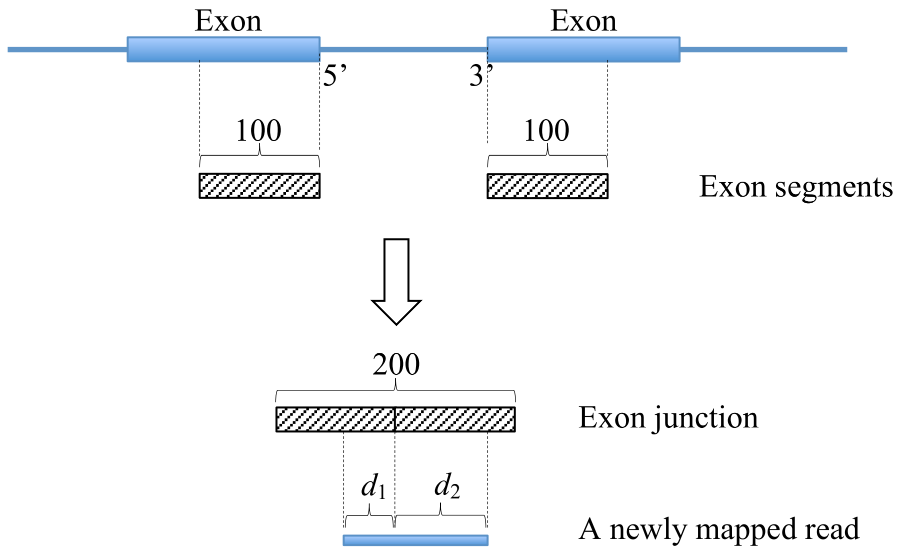

Fig D. Generating exon junctions, and mapping reads to an exon junction. Minimum distance allowed between two exons being joined is 70 bases. Two exon segments adjacent to the joining splice site are extrated, and combined to form an exon junction of length 200 bases. Unmapped reads are mapped against exon junctions. d_1 and d_2 are the number of bases that the read maps to either exon segment. $\min(d_1, d_2) \geq d$ is required for a newly mapped read to be reported from our pipeline. We also calculate the mismatch rate r for each mapped read. d and r are parameters automatically tuned in the range [1, 15] and [0.00, 0.05], respectively, ensuring the most number of calls from the true exon junction library while satisfying no false call from any decoy library.

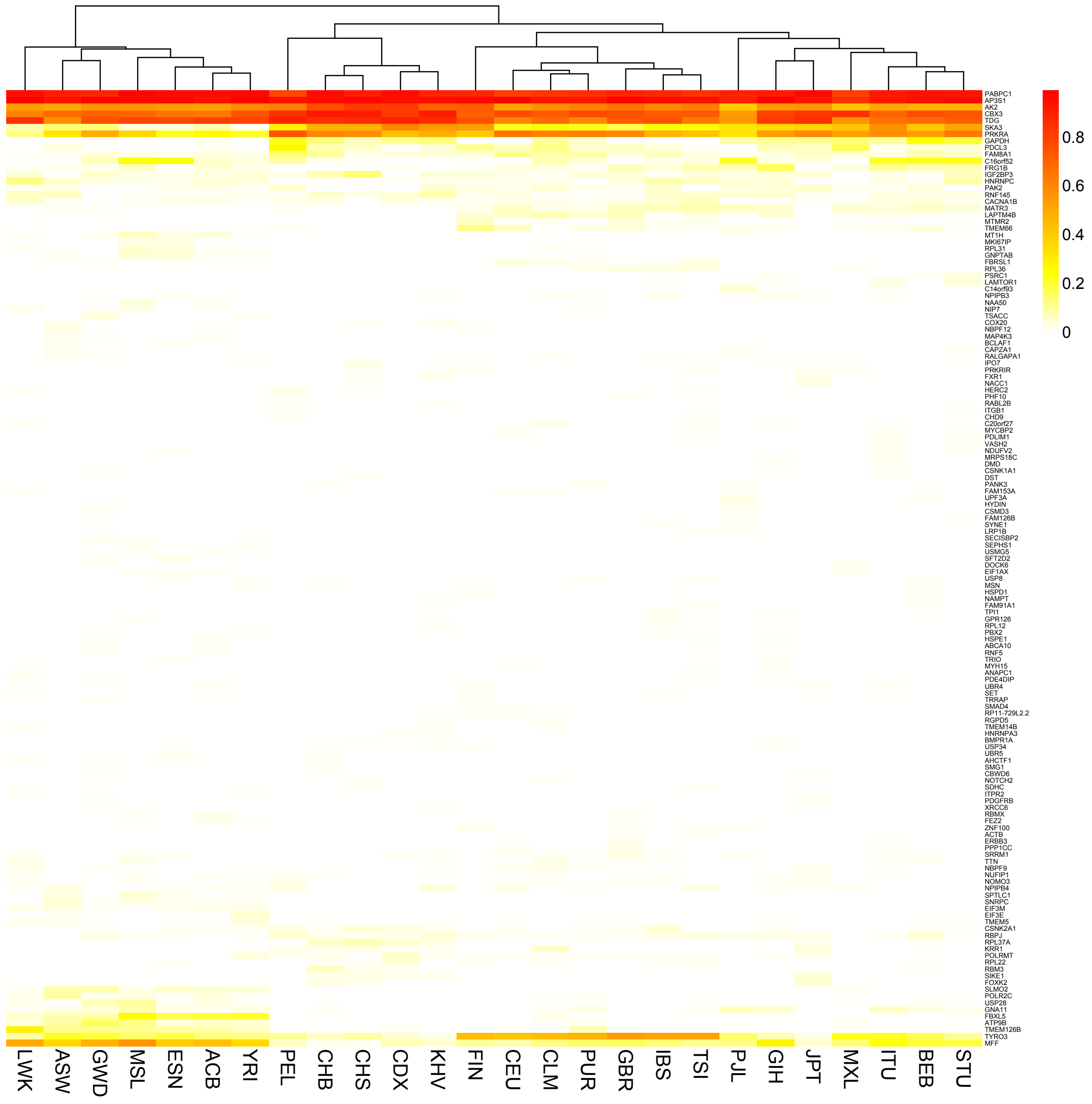

Fig E. Exploration of 142 retroduplication events based on frequency spectrum. Frequency spectrum of 142 retroduplication events that are detected in at least two populations. Hierarchical clustering was used.

Parent gene is hom, the retrodup is…

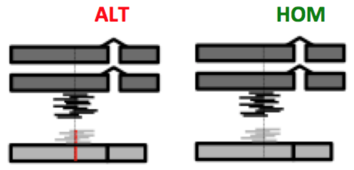

If the depth is …	10X	50X	100X
No retrodup	99.1%	>99.9%	>99.9%
Alt. retrodup	6.76%	0.03%	<0.01%
Ref. retrodup	99.7%	>99.9%	>99.9%

Parent gene is alt, the retrodup is…

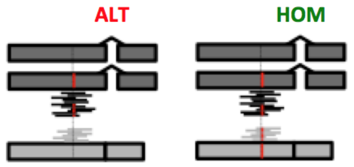

If the depth is …	10X	50X	100X
No retrodup	89.1%	99.3%	>99.9%
Alt. retrodup	59.7%	57.5%	55.4%
Ref. retrodup	92.9%	>99.9%	>99.9%

Fig F. A simplified model of retroduplications affecting SNP calling. We first define the likelihood functions for three genotypes: Pois(n, depth*(1-e)) (AA), Binom(n, depth, 0.5) (AB) and Pois(n, depth*e) (BB). The error rate e = 0.01. We generate all reads from a Poisson distribution with an error rate of 0.01. For genotyping, we picked the one with the highest likelihood and compare it with the ground truth to derive the accuracy as shown in the table. HOM/hom: homozygous/reference allele; ALT/alt: alternative allele.

Table A. Update of Phase 3 retroduplication identifications from Phase 1. In our previous study (Abyzov et al., 2013), we identified retroduplications from the 1000 Genomes Project Phase 1 low-coverage whole-genome sequencing data. In the new study, we identified retroduplications from Phase 3 high-coverage sequencing data. Update of this study is summarized in the table.

	Previous study (Abyzov et al., 2013)	New study update
**Detection of novel retroduplications using exon junction libraries**		
Data	Low-coverage whole-genome sequencing (WGS)	ILLUMINA high-coverage whole-exome sequencing (WES)
Populations	986 WGS of 14 populations + 2 WGS trios (YRI trio, CEU trio)	2,535 WES of 26 populations
Number of retroduplication calls per person	On average 6-10	Median: 6, IQR: 2
Number of unique genes with novel exon junctions	147	503
Outlier individuals	6 JPT individuals	1 CEU individual and 1 LWK individual
**Insertion site detection using mapping of paired end reads**		
Data	968 WGS	2,535 WGS
Number of insertion sites detected	36 unique genes	152 unique genes

Table B. Population differentiation measure of each retroduplication parent gene. Observed F_{ST} is the observed test statistic from the true populations set. The empirical p-value is calculated from permutations tests, comparing observed F_{ST} with the null distribution of F_{ST} derived from 1000 fake population sets. The parent genes are assorted with ascending BH (Benjamini-Hochberg procedure) adjusted p-value. The top 43 parent genes have BH adjusted p-value < 0.001, and they are significant retroduplication parent genes that can differentiate superpopulations.

	Parent Gene Ensembl ID	Observed F_{ST}	p-value	Adjusted p-value (BH)
1	ENSG00000092445	0.138278681	0	0
2	ENSG00000168958	0.137309409	0	0
3	ENSG00000165480	0.120808178	0	0
4	ENSG00000118564	0.109889405	0	0
5	ENSG00000171204	0.07846155	0	0
6	ENSG00000111640	0.060451025	0	0
7	ENSG00000185716	0.058092375	0	0
8	ENSG00000180228	0.056955187	0	0
9	ENSG00000166377	0.04989263	0	0
10	ENSG00000115539	0.038428269	0	0
11	ENSG00000197756	0.035297492	0	0
12	ENSG00000149531	0.032854703	0	0
13	ENSG00000101166	0.031702645	0	0
14	ENSG00000004455	0.02975187	0	0
15	ENSG00000122565	0.029516431	0	0
16	ENSG00000139372	0.026314177	0	0
17	ENSG00000015479	0.024405028	0	0
18	ENSG00000048028	0.023831726	0	0
19	ENSG00000070756	0.022783149	0	0
20	ENSG00000087053	0.020313783	0	0
21	ENSG00000052723	0.020214209	0	0
22	ENSG00000133872	0.02011706	0	0
23	ENSG00000104341	0.017653856	0	0
24	ENSG00000102978	0.017058594	0	0
25	ENSG00000141568	0.015530902	0	0
26	ENSG00000205358	0.014956016	0	0
27	ENSG00000088256	0.014498964	0	0
28	ENSG00000111615	0.013778157	0	0
29	ENSG00000149100	0.013289565	0	0
30	ENSG00000149357	0.012697414	0	0
31	ENSG00000102317	0.01203019	0	0
32	ENSG00000130255	0.010837415	0	0
33	ENSG00000111670	0.010644515	0	0

34	ENSG00000137414	0.010048388	0	0
35	ENSG00000092199	0.009994358	0	0
36	ENSG00000090054	0.009955327	0	0
37	ENSG00000124562	0.009955327	0	0
38	ENSG00000177879	0.008665282	0	0
39	ENSG00000100802	0.008163628	0	0
40	ENSG00000101444	0.008163628	0	0
41	ENSG00000134222	0.008163628	0	0
42	ENSG00000104408	0.006629006	0	0
43	ENSG00000112787	0.006022602	0	0
44	ENSG00000071082	0.009566276	0.001	0.010934783
45	ENSG00000118600	0.007736901	0.001	0.010934783
46	ENSG00000120438	0.006629006	0.001	0.010934783
47	ENSG00000114416	0.007750092	0.002	0.021404255
48	ENSG00000121579	0.005521986	0.004	0.041061224
49	ENSG00000155438	0.005521986	0.004	0.041061224
50	ENSG00000160877	0.006197622	0.005	0.0503
51	ENSG00000163467	0.005521986	0.006	0.059176471
52	ENSG00000136231	0.0052821	0.008	0.075924528
53	ENSG00000163319	0.004894305	0.008	0.075924528
54	ENSG00000169062	0.004894305	0.009	0.083833333
55	ENSG00000197020	0.004789575	0.011	0.098803571
56	ENSG00000145860	0.004779859	0.011	0.098803571
57	ENSG00000176915	0.004894305	0.013	0.114719298
58	ENSG00000186298	0.004773668	0.016	0.138758621
59	ENSG00000156467	0.004896106	0.017	0.144932203
60	ENSG00000099821	0.004572527	0.018	0.14842623
61	ENSG00000116251	0.004114264	0.018	0.14842623
62	ENSG00000132603	0.004352587	0.022	0.178483871
63	ENSG00000171055	0.003310573	0.029	0.231539683
64	ENSG00000213064	0.003310573	0.037	0.290796875
65	ENSG00000187742	0.003310573	0.04	0.309538462
66	ENSG00000077809	0.003261581	0.042	0.320090909
67	ENSG00000136144	0.003310573	0.043	0.322820896
68	ENSG00000011566	0.003310573	0.044	0.325470588
69	ENSG00000166454	0.003310573	0.047	0.342623188
70	ENSG00000173915	0.003310573	0.05	0.359285714
71	ENSG00000107438	0.003263524	0.055	0.389647887
72	ENSG00000155744	0.003261581	0.056	0.391222222
73	ENSG00000182899	0.003261581	0.063	0.43409589
74	ENSG00000198947	0.003261581	0.064	0.435027027

75	ENSG00000204628	0.003261581	0.065	0.435933333
76	ENSG00000143494	0.003261581	0.067	0.443434211
77	ENSG00000137492	0.003223297	0.072	0.470337662
78	ENSG00000156795	0.003181188	0.084	0.541692308
79	ENSG00000101266	0.003062007	0.098	0.611082645
80	ENSG00000141646	0.003181188	0.106	0.611082645
81	ENSG00000267699	0.003181188	0.106	0.611082645
82	ENSG00000075624	0.003181188	0.108	0.611082645
83	ENSG00000203667	0.003169033	0.11	0.611082645
84	ENSG00000203943	0.002447086	0.116	0.611082645
85	ENSG00000204790	0.003096362	0.12	0.611082645
86	ENSG00000167004	0.003096362	0.124	0.611082645
87	ENSG00000131732	0.002447086	0.124	0.611082645
88	ENSG00000066027	0.002447086	0.126	0.611082645
89	ENSG00000185842	0.002447086	0.126	0.611082645
90	ENSG00000123545	0.002447086	0.13	0.611082645
91	ENSG00000197930	0.002447086	0.13	0.611082645
92	ENSG00000169139	0.002447086	0.131	0.611082645
93	ENSG00000089157	0.002447086	0.132	0.611082645
94	ENSG00000185305	0.002447086	0.132	0.611082645
95	ENSG00000196419	0.003096362	0.133	0.611082645
96	ENSG00000132692	0.002447086	0.133	0.611082645
97	ENSG00000137601	0.002447086	0.133	0.611082645
98	ENSG00000143341	0.002447086	0.133	0.611082645
99	ENSG00000163492	0.002447086	0.133	0.611082645
100	ENSG00000135457	0.002447086	0.134	0.611082645
101	ENSG00000198554	0.002447086	0.134	0.611082645
102	ENSG00000204262	0.002447086	0.134	0.611082645
103	ENSG00000106636	0.002447086	0.135	0.611082645
104	ENSG00000116001	0.002447086	0.135	0.611082645
105	ENSG00000155313	0.002447086	0.135	0.611082645
106	ENSG00000173674	0.002503289	0.137	0.611082645
107	ENSG00000100030	0.002447086	0.137	0.611082645
108	ENSG00000113013	0.002447086	0.137	0.611082645
109	ENSG00000188647	0.002447086	0.137	0.611082645
110	ENSG00000165898	0.002447086	0.138	0.611082645
111	ENSG00000118113	0.002447086	0.139	0.611082645
112	ENSG00000164253	0.002447086	0.139	0.611082645
113	ENSG00000078304	0.002447086	0.14	0.611082645
114	ENSG00000004866	0.002447086	0.142	0.611082645
115	ENSG00000125144	0.002447086	0.142	0.611082645

116	ENSG00000153956	0.002447086	0.142	0.611082645
117	ENSG00000145730	0.002447086	0.143	0.611082645
118	ENSG00000130159	0.002447086	0.144	0.611082645
119	ENSG00000163877	0.002447086	0.146	0.611082645
120	ENSG00000164032	0.002447086	0.146	0.611082645
121	ENSG00000185261	0.002447086	0.147	0.611082645
122	ENSG00000149273	0.003096362	0.149	0.613414634
123	ENSG00000146776	0.002447086	0.15	0.613414634
124	ENSG00000170144	0.003096362	0.152	0.616580645
125	ENSG00000153391	0.003096362	0.155	0.618769841
126	ENSG00000025156	0.002447086	0.155	0.618769841
127	ENSG00000005810	0.002178645	0.157	0.621818898
128	ENSG00000170906	0.002447086	0.16	0.62875
129	ENSG00000133818	0.002447086	0.167	0.651170543
130	ENSG00000178104	0.002206176	0.197	0.760259542
131	ENSG00000086475	0.002206176	0.198	0.760259542
132	ENSG00000147274	0.002288754	0.2	0.762007463
133	ENSG00000029363	0.002206176	0.202	0.762007463
134	ENSG00000204308	0.002206176	0.203	0.762007463
135	ENSG00000065361	0.002138433	0.206	0.767540741
136	ENSG00000120137	0.002256828	0.209	0.772992647
137	ENSG00000111669	0.002138433	0.216	0.786073593
138	ENSG00000154263	0.002206176	0.218	0.786073593
139	ENSG00000128731	0.002135409	0.243	0.786073593
140	ENSG00000205339	0.002229863	0.246	0.786073593
141	ENSG00000107779	0.00208186	0.248	0.786073593
142	ENSG00000150093	0.001644321	0.264	0.786073593
143	ENSG00000150760	0.001630146	0.293	0.786073593
144	ENSG00000166743	0.001630146	0.293	0.786073593
145	ENSG00000143436	0.001630146	0.298	0.786073593
146	ENSG00000110700	0.001630146	0.301	0.786073593
147	ENSG00000163638	0.001630146	0.301	0.786073593
148	ENSG00000077380	0.001630146	0.309	0.786073593
149	ENSG00000133112	0.001630146	0.309	0.786073593
150	ENSG00000181163	0.001630146	0.309	0.786073593
151	ENSG00000148408	0.001858721	0.311	0.786073593
152	ENSG00000196586	0.001630146	0.312	0.786073593
153	ENSG00000198420	0.001630146	0.313	0.786073593
154	ENSG00000157856	0.001630146	0.314	0.786073593
155	ENSG00000118307	0.001630146	0.315	0.786073593
156	ENSG00000130024	0.001624223	0.315	0.786073593

157	ENSG00000112379	0.001630146	0.316	0.786073593
158	ENSG00000213741	0.001630146	0.316	0.786073593
159	ENSG00000064666	0.001630146	0.317	0.786073593
160	ENSG00000171502	0.001630146	0.317	0.786073593
161	ENSG00000243716	0.001630146	0.317	0.786073593
162	ENSG00000177200	0.001624223	0.317	0.786073593
163	ENSG00000089472	0.001630146	0.32	0.786073593
164	ENSG00000157483	0.001630146	0.32	0.786073593
165	ENSG00000048544	0.001630146	0.321	0.786073593
166	ENSG00000174827	0.001630146	0.321	0.786073593
167	ENSG00000100083	0.001630146	0.322	0.786073593
168	ENSG00000101367	0.001630146	0.322	0.786073593
169	ENSG00000116489	0.002016079	0.323	0.786073593
170	ENSG00000115760	0.001630146	0.324	0.786073593
171	ENSG00000198648	0.001630146	0.324	0.786073593
172	ENSG00000213585	0.001630146	0.326	0.786073593
173	ENSG00000003402	0.001630146	0.327	0.786073593
174	ENSG00000049759	0.001630146	0.327	0.786073593
175	ENSG00000116497	0.001630146	0.327	0.786073593
176	ENSG00000116809	0.001630146	0.327	0.786073593
177	ENSG00000134333	0.001630146	0.327	0.786073593
178	ENSG00000152208	0.001630146	0.327	0.786073593
179	ENSG00000161203	0.001630146	0.327	0.786073593
180	ENSG00000119335	0.001962463	0.328	0.786073593
181	ENSG00000054523	0.001630146	0.328	0.786073593
182	ENSG00000185088	0.001630146	0.328	0.786073593
183	ENSG00000174093	0.001630146	0.329	0.786073593
184	ENSG00000178127	0.002016079	0.33	0.786073593
185	ENSG00000254685	0.001630146	0.33	0.786073593
186	ENSG00000145147	0.001630146	0.331	0.786073593
187	ENSG00000114867	0.001630146	0.332	0.786073593
188	ENSG00000133056	0.001630146	0.333	0.786073593
189	ENSG00000120658	0.001630146	0.334	0.786073593
190	ENSG00000132694	0.001630146	0.334	0.786073593
191	ENSG00000145216	0.001630146	0.334	0.786073593
192	ENSG00000163312	0.001630146	0.334	0.786073593
193	ENSG00000163389	0.001630146	0.334	0.786073593
194	ENSG00000165521	0.001630146	0.334	0.786073593
195	ENSG00000198848	0.001630146	0.334	0.786073593
196	ENSG00000241973	0.001630146	0.334	0.786073593
197	ENSG00000243678	0.001630146	0.334	0.786073593

198	ENSG00000101132	0.001630146	0.335	0.786073593
199	ENSG00000184445	0.001630146	0.335	0.786073593
200	ENSG00000139144	0.001630146	0.337	0.786073593
201	ENSG00000188641	0.001630146	0.337	0.786073593
202	ENSG00000081026	0.001630146	0.338	0.786073593
203	ENSG00000122643	0.001630146	0.338	0.786073593
204	ENSG00000157423	0.002016079	0.339	0.786073593
205	ENSG00000074219	0.001630146	0.339	0.786073593
206	ENSG00000072818	0.001630146	0.34	0.786073593
207	ENSG00000082516	0.001630146	0.34	0.786073593
208	ENSG00000135837	0.001630146	0.34	0.786073593
209	ENSG00000136895	0.001630146	0.34	0.786073593
210	ENSG00000133962	0.001630146	0.341	0.786073593
211	ENSG00000163029	0.001630146	0.341	0.786073593
212	ENSG00000181929	0.001630146	0.342	0.786073593
213	ENSG00000196712	0.001630146	0.342	0.786073593
214	ENSG00000213759	0.001630146	0.342	0.786073593
215	ENSG00000116748	0.001630146	0.344	0.786073593
216	ENSG00000123505	0.001630146	0.344	0.786073593
217	ENSG00000007392	0.001630146	0.345	0.786073593
218	ENSG00000109270	0.001630146	0.346	0.786073593
219	ENSG00000198586	0.001630146	0.346	0.786073593
220	ENSG00000143515	0.001630146	0.349	0.786073593
221	ENSG00000197136	0.001630146	0.349	0.786073593
222	ENSG00000079805	0.001630146	0.356	0.786073593
223	ENSG00000085788	0.001630146	0.358	0.786073593
224	ENSG00000142609	0.001630146	0.358	0.786073593
225	ENSG00000165995	0.001630146	0.358	0.786073593
226	ENSG00000173812	0.001630146	0.358	0.786073593
227	ENSG00000187323	0.001630146	0.358	0.786073593
228	ENSG00000077522	0.001630146	0.36	0.786073593
229	ENSG00000137714	0.001630146	0.36	0.786073593
230	ENSG00000183914	0.001630146	0.36	0.786073593
231	ENSG00000134250	0.001603016	0.361	0.786073593
232	ENSG00000138792	0.001630146	0.364	0.789189655
233	ENSG00000079974	0.001603016	0.373	0.80523176
234	ENSG00000015568	0.001603016	0.387	0.831884615
235	ENSG00000168214	0.001613257	0.408	0.873293617
236	ENSG00000180370	0.001505758	0.416	0.886644068
237	ENSG00000130158	0.00138047	0.444	0.917725166
238	ENSG00000168614	0.001532636	0.45	0.917725166

239	ENSG00000186275	0.001474764	0.491	0.917725166
240	ENSG00000101220	0.001458378	0.493	0.917725166
241	ENSG00000168028	0.001589966	0.502	0.917725166
242	ENSG00000156802	0.001589966	0.504	0.917725166
243	ENSG00000144381	0.001215591	0.506	0.917725166
244	ENSG00000083857	0.001589966	0.508	0.917725166
245	ENSG00000170242	0.001589966	0.508	0.917725166
246	ENSG00000174799	0.001589966	0.512	0.917725166
247	ENSG00000180777	0.001589966	0.514	0.917725166
248	ENSG00000257103	0.001589966	0.516	0.917725166
249	ENSG00000042781	0.001589966	0.517	0.917725166
250	ENSG00000066813	0.001589966	0.517	0.917725166
251	ENSG00000131018	0.001215591	0.518	0.917725166
252	ENSG00000118965	0.001589966	0.519	0.917725166
253	ENSG00000168702	0.001215591	0.519	0.917725166
254	ENSG00000135976	0.001589966	0.52	0.917725166
255	ENSG00000136888	0.001589966	0.52	0.917725166
256	ENSG00000152558	0.001589966	0.52	0.917725166
257	ENSG00000056097	0.001589966	0.523	0.917725166
258	ENSG00000086758	0.001589966	0.523	0.917725166
259	ENSG00000122512	0.001589966	0.523	0.917725166
260	ENSG00000112282	0.001589966	0.524	0.917725166
261	ENSG00000171314	0.001589966	0.524	0.917725166
262	ENSG00000197728	0.001589966	0.524	0.917725166
263	ENSG00000123104	0.001460621	0.524	0.917725166
264	ENSG00000117245	0.001589966	0.525	0.917725166
265	ENSG00000183354	0.001589966	0.525	0.917725166
266	ENSG00000165084	0.001589966	0.526	0.917725166
267	ENSG00000166747	0.001589966	0.526	0.917725166
268	ENSG00000186998	0.001589966	0.526	0.917725166
269	ENSG00000114857	0.001589966	0.527	0.917725166
270	ENSG00000147050	0.001589966	0.527	0.917725166
271	ENSG00000153201	0.001589966	0.528	0.917725166
272	ENSG00000176853	0.001215591	0.529	0.917725166
273	ENSG00000112706	0.001589966	0.53	0.917725166
274	ENSG00000139218	0.001589966	0.531	0.917725166
275	ENSG00000140992	0.001589966	0.531	0.917725166
276	ENSG00000152348	0.001589966	0.532	0.917725166
277	ENSG00000053108	0.001589966	0.533	0.917725166
278	ENSG00000143702	0.001589966	0.533	0.917725166
279	ENSG00000150459	0.001589966	0.533	0.917725166

280	ENSG00000149311	0.001589966	0.534	0.917725166
281	ENSG00000111247	0.001589966	0.535	0.917725166
282	ENSG00000197006	0.001589966	0.535	0.917725166
283	ENSG00000119685	0.001589966	0.536	0.917725166
284	ENSG00000134453	0.001589966	0.536	0.917725166
285	ENSG00000198626	0.001589966	0.537	0.917725166
286	ENSG00000115839	0.001589966	0.538	0.917725166
287	ENSG00000155561	0.001589966	0.538	0.917725166
288	ENSG00000165181	0.001589966	0.538	0.917725166
289	ENSG00000165899	0.001589966	0.538	0.917725166
290	ENSG00000083290	0.001589966	0.54	0.917725166
291	ENSG00000132570	0.001589966	0.54	0.917725166
292	ENSG00000183091	0.001589966	0.541	0.917725166
293	ENSG00000154447	0.001589966	0.542	0.917725166
294	ENSG00000116678	0.001589966	0.543	0.917725166
295	ENSG00000157388	0.001589966	0.545	0.917725166
296	ENSG00000144821	0.001215591	0.545	0.917725166
297	ENSG00000177600	0.001589966	0.548	0.917725166
298	ENSG00000143207	0.001589966	0.549	0.917725166
299	ENSG00000103495	0.001589966	0.55	0.917725166
300	ENSG00000198399	0.001589966	0.55	0.917725166
301	ENSG00000154059	0.001589966	0.551	0.917725166
302	ENSG00000153207	0.001460621	0.551	0.917725166
303	ENSG00000162927	0.001589966	0.555	0.921336634
304	ENSG00000103657	0.001589966	0.559	0.924924342
305	ENSG00000264364	0.001589966	0.564	0.929491909
306	ENSG00000169499	0.001589966	0.567	0.929491909
307	ENSG00000157350	0.001589966	0.568	0.929491909
308	ENSG00000185864	0.001215934	0.57	0.929491909
309	ENSG00000169246	0.001203715	0.571	0.929491909
310	ENSG00000151914	0.001194385	0.605	0.98166129
311	ENSG00000105835	0.001194385	0.612	0.989826367
312	ENSG00000143252	0.001174287	0.678	0.999534704
313	ENSG00000197958	0.001174287	0.688	0.999534704
314	ENSG00000140319	0.00154757	0.712	0.999534704
315	ENSG00000134970	0.00154757	0.714	0.999534704
316	ENSG00000146574	0.00154757	0.714	0.999534704
317	ENSG00000243414	0.00154757	0.714	0.999534704
318	ENSG00000251201	0.00154757	0.714	0.999534704
319	ENSG00000163605	0.00154757	0.716	0.999534704
320	ENSG00000164118	0.00154757	0.716	0.999534704

321	ENSG00000170160	0.00154757	0.717	0.999534704
322	ENSG00000047457	0.00154757	0.718	0.999534704
323	ENSG00000128699	0.00154757	0.718	0.999534704
324	ENSG00000197563	0.00154757	0.718	0.999534704
325	ENSG00000141552	0.00154757	0.719	0.999534704
326	ENSG00000121741	0.00154757	0.723	0.999534704
327	ENSG00000139579	0.00154757	0.723	0.999534704
328	ENSG00000146350	0.00154757	0.724	0.999534704
329	ENSG00000188906	0.00154757	0.724	0.999534704
330	ENSG00000096063	0.00154757	0.725	0.999534704
331	ENSG00000066933	0.00154757	0.726	0.999534704
332	ENSG00000156374	0.00154757	0.727	0.999534704
333	ENSG00000167460	0.00154757	0.728	0.999534704
334	ENSG00000118997	0.00154757	0.731	0.999534704
335	ENSG00000126787	0.00154757	0.731	0.999534704
336	ENSG00000136810	0.00154757	0.733	0.999534704
337	ENSG00000138688	0.00154757	0.733	0.999534704
338	ENSG00000205359	0.00154757	0.733	0.999534704
339	ENSG00000124789	0.00154757	0.734	0.999534704
340	ENSG00000175387	0.00154757	0.734	0.999534704
341	ENSG00000042317	0.00154757	0.735	0.999534704
342	ENSG00000147679	0.00154757	0.735	0.999534704
343	ENSG00000140157	0.00154757	0.737	0.999534704
344	ENSG00000165732	0.00154757	0.737	0.999534704
345	ENSG00000213995	0.00154757	0.737	0.999534704
346	ENSG00000008128	0.00154757	0.738	0.999534704
347	ENSG00000035141	0.00154757	0.738	0.999534704
348	ENSG00000090402	0.00154757	0.738	0.999534704
349	ENSG00000104413	0.00154757	0.738	0.999534704
350	ENSG00000152818	0.00154757	0.738	0.999534704
351	ENSG00000160075	0.00154757	0.738	0.999534704
352	ENSG00000158321	0.00154757	0.739	0.999534704
353	ENSG00000060718	0.00154757	0.74	0.999534704
354	ENSG00000147403	0.00154757	0.74	0.999534704
355	ENSG00000122008	0.00154757	0.742	0.999534704
356	ENSG00000152484	0.00154757	0.742	0.999534704
357	ENSG00000124721	0.00154757	0.743	0.999534704
358	ENSG00000167721	0.00154757	0.743	0.999534704
359	ENSG00000127980	0.00154757	0.744	0.999534704
360	ENSG00000104613	0.00154757	0.745	0.999534704
361	ENSG00000127184	0.00154757	0.746	0.999534704

362	ENSG00000205726	0.00154757	0.746	0.999534704
363	ENSG00000115541	0.000921411	0.746	0.999534704
364	ENSG00000163754	0.00154757	0.748	0.999534704
365	ENSG00000188219	0.00154757	0.748	0.999534704
366	ENSG00000023228	0.00154757	0.75	0.999534704
367	ENSG00000145592	0.00154757	0.75	0.999534704
368	ENSG00000163554	0.00154757	0.75	0.999534704
369	ENSG00000137312	0.00154757	0.751	0.999534704
370	ENSG00000151067	0.00154757	0.751	0.999534704
371	ENSG00000068878	0.00154757	0.752	0.999534704
372	ENSG00000134287	0.00154757	0.752	0.999534704
373	ENSG00000143543	0.00154757	0.752	0.999534704
374	ENSG00000163539	0.00154757	0.752	0.999534704
375	ENSG00000272822	0.00154757	0.752	0.999534704
376	ENSG00000100316	0.00154757	0.753	0.999534704
377	ENSG00000128585	0.00154757	0.753	0.999534704
378	ENSG00000110200	0.00154757	0.755	0.999534704
379	ENSG00000078114	0.00154757	0.758	0.999534704
380	ENSG00000122966	0.00154757	0.759	0.999534704
381	ENSG00000180694	0.00154757	0.759	0.999534704
382	ENSG00000174469	0.00154757	0.76	0.999534704
383	ENSG00000103226	0.000823909	0.762	0.999534704
384	ENSG00000038382	0.000971839	0.764	0.999534704
385	ENSG00000136068	0.00154757	0.768	0.999534704
386	ENSG00000113712	0.000971839	0.769	0.999534704
387	ENSG00000137210	0.000909932	0.771	0.999534704
388	ENSG00000163510	0.00154757	0.772	0.999534704
389	ENSG00000133226	0.000777806	0.773	0.999534704
390	ENSG00000164796	0.000971839	0.792	1
391	ENSG00000115464	0.00079997	0.82	1
392	ENSG00000138592	0.0005947	0.876	1
393	ENSG00000155657	0.000504163	0.877	1
394	ENSG00000204304	0.00095174	0.879	1
395	ENSG00000174373	0.0005947	0.881	1
396	ENSG00000170074	0.000508231	0.881	1
397	ENSG00000153107	0.000651547	0.884	1
398	ENSG00000196367	0.00095174	0.891	1
399	ENSG00000112414	0.00095174	0.91	1
400	ENSG00000083635	0.000422222	0.913	1
401	ENSG00000147065	0.000637404	0.934	1
402	ENSG00000001629	0.001102653	1	1

403	ENSG00000004897	0.001102653	1	1
404	ENSG00000007402	0.001102653	1	1
405	ENSG00000008405	0.001102653	1	1
406	ENSG00000029534	0.001102653	1	1
407	ENSG00000039139	0.001102653	1	1
408	ENSG00000058262	0.001102653	1	1
409	ENSG00000065809	0.001102653	1	1
410	ENSG00000069248	0.001102653	1	1
411	ENSG00000077097	0.001102653	1	1
412	ENSG00000081237	0.001102653	1	1
413	ENSG00000081479	0.001102653	1	1
414	ENSG00000083312	0.001102653	1	1
415	ENSG00000084774	0.001102653	1	1
416	ENSG00000088280	0.001102653	1	1
417	ENSG00000099977	0.001102653	1	1
418	ENSG00000100075	0.001102653	1	1
419	ENSG00000102078	0.001102653	1	1
420	ENSG00000105193	0.001102653	1	1
421	ENSG00000106028	0.001102653	1	1
422	ENSG00000108518	0.001102653	1	1
423	ENSG00000111300	0.001102653	1	1
424	ENSG00000111752	0.001102653	1	1
425	ENSG00000112159	0.001102653	1	1
426	ENSG00000112200	0.001102653	1	1
427	ENSG00000117758	0.001102653	1	1
428	ENSG00000118454	0.001102653	1	1
429	ENSG00000118816	0.001102653	1	1
430	ENSG00000121289	0.001102653	1	1
431	ENSG00000121570	0.001102653	1	1
432	ENSG00000121671	0.001102653	1	1
433	ENSG00000122707	0.001102653	1	1
434	ENSG00000124702	0.001102653	1	1
435	ENSG00000124920	0.001102653	1	1
436	ENSG00000125445	0.001102653	1	1
437	ENSG00000125459	0.001102653	1	1
438	ENSG00000125952	0.001102653	1	1
439	ENSG00000128641	0.001102653	1	1
440	ENSG00000129566	0.001102653	1	1
441	ENSG00000129675	0.001102653	1	1
442	ENSG00000132122	0.001102653	1	1
443	ENSG00000132522	0.001102653	1	1

444	ENSG00000132676	0.001102653	1	1
445	ENSG00000132819	0.001102653	1	1
446	ENSG00000134109	0.001102653	1	1
447	ENSG00000134627	0.001102653	1	1
448	ENSG00000135596	0.001102653	1	1
449	ENSG00000136011	0.001102653	1	1
450	ENSG00000136104	0.001102653	1	1
451	ENSG00000137700	0.001102653	1	1
452	ENSG00000138448	0.001102653	1	1
453	ENSG00000138778	0.001102653	1	1
454	ENSG00000140853	0.001102653	1	1
455	ENSG00000140905	0.001102653	1	1
456	ENSG00000143452	0.001102653	1	1
457	ENSG00000145741	0.001102653	1	1
458	ENSG00000148356	0.001102653	1	1
459	ENSG00000149547	0.001102653	1	1
460	ENSG00000150527	0.001102653	1	1
461	ENSG00000150787	0.001102653	1	1
462	ENSG00000151422	0.001102653	1	1
463	ENSG00000151779	0.001102653	1	1
464	ENSG00000152601	0.001102653	1	1
465	ENSG00000154265	0.001102653	1	1
466	ENSG00000160188	0.001102653	1	1
467	ENSG00000160211	0.001102653	1	1
468	ENSG00000162869	0.001102653	1	1
469	ENSG00000164190	0.001102653	1	1
470	ENSG00000165390	0.001102653	1	1
471	ENSG00000165912	0.001102653	1	1
472	ENSG00000167548	0.001102653	1	1
473	ENSG00000168778	0.001102653	1	1
474	ENSG00000171132	0.001102653	1	1
475	ENSG00000172115	0.001102653	1	1
476	ENSG00000175582	0.001102653	1	1
477	ENSG00000176783	0.001102653	1	1
478	ENSG00000177156	0.001102653	1	1
479	ENSG00000182095	0.001102653	1	1
480	ENSG00000182621	0.001102653	1	1
481	ENSG00000183765	0.001102653	1	1
482	ENSG00000186439	0.001102653	1	1
483	ENSG00000187240	0.001102653	1	1
484	ENSG00000187522	0.001102653	1	1

485	ENSG00000197121	0.001102653	1	1
486	ENSG00000197386	0.001102653	1	1
487	ENSG00000197893	0.001102653	1	1
488	ENSG00000197969	0.001102653	1	1
489	ENSG00000198355	0.001102653	1	1
490	ENSG00000198382	0.001102653	1	1
491	ENSG00000205038	0.001102653	1	1
492	ENSG00000211460	0.001102653	1	1
493	ENSG00000227345	0.001102653	1	1
494	ENSG00000241186	0.001102653	1	1
495	ENSG00000253729	0.001102653	1	1
496	ENSG00000254999	0.001102653	1	1
497	ENSG00000258941	0.001102653	1	1
498	ENSG00000261915	0.001102653	1	1
499	ENSG00000264424	0.001102653	1	1
500	ENSG00000104517	0.000930534	1	1
501	ENSG00000113721	0.000930534	1	1
502	ENSG00000157106	0.000930534	1	1
503	ENSG00000127481	0.000283085	1	1

Table C. Percentage of total variants (retroduplications, or SNPs) private to each superpopulation. The novel retroduplication set can be found in S2 File. The SNPs were extracted from the 1000 Genomes Project Phase 3 dataset (Auton et al., 2015), available at ftp://ftp.1000genomes.ebi.ac.uk/vol1/ftp/release/20130502/ALL.wgs.phase3_shapeit2_mvncall_integrated_v5b.20130502.sites.vcf.gz.

	**Total variant sites detected in all genomes**	**Total variant sites private to a superpopulation**	**AFR**	**AMR**	**EAS**	**EUR**	**SAS**
Retroduplications	503	397<br>(78.9%)	119<br>(23.7%)	39<br>(7.8%)	80<br>(15.9%)	65<br>(12.9%)	94<br>(18.7%)
SNPs	81,443,073	55,983,609<br>(68.7%)	20,120,050<br>(24.7%)	5,577,247<br>(6.8%)	11,742,915<br>(14.4%)	7,189,248<br>(8.8%)	11,354,149<br>(13.9%)

Table D. Disease-associated retroduplication insertions. These insertion target (inserted) genes associated with diseases are supported by literature, mined from the DISEASES database.

Insertion site chr.	Insertion site start	Insertion site end	Parent gene name	Parent gene Ensembl ID	Parent gene chr.	Parent gene start	Parent gene end	Affected population (by insertion points)	Inserted gene name	Ref	Disease, evidence
chr19	13331914	13332160	ANK1	ENSG00000029534	chr8	41510738	41754280	LWK	CACNA1A	DOID:0060178	Familial,hemiplegic,migraine,GHR,CURATED
									CACNA1A	DOID:1289	Neurodegenerative,disease,UniProtKB-KW,CURATED
									CACNA1A	DOID:1441	Spinocerebellar,ataxia,UniProtKB-KW,CURATED
									CACNA1A	DOID:963	Episodic,ataxia,GHR,CURATED
chr3	8868980	8869077	SLMO2	ENSG00000101166	chr20	57608199	57617964	ACB,ESN,GWD,YRI	CAV3	DOID:0050700	Cardiomyopathy,UniProtKB-KW,CURATED,
									CAV3	DOID:11724	limb-girdle,muscular,dystrophy,GHR,CURATED
									CAV3	DOID:11724	limb-girdle,muscular,dystrophy,UniProtKB-KW,CURATED
									CAV3	DOID:2843	Long,QT,syndrome,UniProtKB-KW,CURATED
chr8	68377448	68377749	TPI1	ENSG00000111669	chr12	6976282	6980112	GIH	CPA6	DOID:1826	Epilepsy,syndrome,UniProtKB-KW,CURATED
chr2	116376651	116376659	SET	ENSG00000119335	chr9	131445702	131458679	CEU,FIN,IBS,LWK	DPP10	DOID:2841	Asthma,UniProtKB-KW,CURATED,
chr15	67871200	67871563	COX7C	ENSG00000127184	chr5	85913720	85916779	CHS	MAP2K5	DOID:0050425	Restless,legs,syndrome,GHR,CURATED
chr10	30651932	30652023	ITSN2	ENSG00000198399	chr2	24425732	24583583	CEU	MTPAP	DOID:1289	Neurodegenerative,disease,UniProtKB-KW,CURATED
chr5	149311980	149312306	MFF	ENSG00000168958	chr2	228189866	228222550	YRI	PDE6A	DOID:10584	Retinitis,pigmentosa,GHR,CURATED
									PDE6A	DOID:10584	Retinitis,pigmentosa,UniProtKB-KW,CURATED
chr6	167114767	167114812	IGF2BP3	ENSG00000136231	chr7	23349827	23510086	BEB,CLM,GWD,JPT,YRI	RPS6KA2	DOID:162	Cancer,UniProtKB-KW,CURATED,
chr6	167114167	167114249	IGF2BP3	ENSG00000136231	chr7	23349827	23510086	CHB,ESN,IBS,LWK,MSL,PUR,YRI			,,,,
chr6	167113886	167113963	IGF2BP3	ENSG00000136231	chr7	23349827	23510086	PJL			,,,,
chr13	36934280	36934427	RALGAPA1	ENSG00000174373	chr14	36007557	36278510	ASW,IBS	SPG20	DOID:1289	Neurodegenerative,disease,UniProtKB-KW,CURATED
									SPG20	DOID:2476	Hereditary,spastic,paraplegia,UniProtKB-KW,CURATED
chrX	41032571	41032679	PFN1	ENSG00000108518	chr17	4848946	4852356	GWD	USP9X	DOID:1059	Intellectual,disability,UniProtKB-KW,CURATED

Table E. Exon-exon junction calls from two trios. Comparison of the number of calls from different platforms.

	CEU trio	YRI trio
High-coverage WGS	13	11
High-coverage WES	11	7
Shallow WGS	6 (NA12878 only, parents' data not available)	5

Estimation of FDR in real data:

Our method could take advantage of the high-coverage WES. In the previous study (Abyzov et al. 2013), retroduplications were discovered per population (i.e. all the individuals in the same population were pooled), since the coverage of Phase 1 WGS data was shallow. By analyzing the pooled shallow WGS data, there was less than 1 novel retroduplication discovered per individual. In comparison, here in this study, we identified novel retroduplications individually using high-coverage WES data, and the number of detected novel retroduplications per individual (median: 6) is significantly more than in the previous study.

Besides, Abyzov et al. also called retroduplications in two deep sequenced trios. These retroduplications have been verified both computationally and experimentally in Abyzov et al. In the CEU and YRI trios, they identified 13 and 11 novel retroduplications, respectively. The numbers are comparable to what we found in our study (median: 6 per individual). We would expect the sensitivity of our WES-based method lie between low-coverage and high-coverage WGS-based method. Indeed, we identified 11 and 7 unique retroduplications in CEU and YRI trios, which are slightly less than 13 and 11.

Furthermore, among the 6 retroduplications that we identified by exon-exon junction strategy on high-coverage WES in the CEU child (NA12878), 4 of them (SKA3, TDG, CBX3 and AP3S1) were also identified by Abyzov et al. using high-coverage WGS data but not AK2 and HNRNPC. In the YRI trio, 5/7 overlapped with the set reported by Abyzov et al. (overlapped: TMEM5, CBX3, ATP9B, MFF and AP3S1, not overlapped: PABPC1 and AK2)." But AK2 and PABPC1 are in the common retroduplication set that Abyzov et al. reported. Taken all together, our estimation of FDR using a validated, independent set is 7.7% (1/13). Noticeably, this is likely an underestimated as some of our unique retroduplications might be missed in Abyzov et al.
